# Synthesis of new Pro-PYE ligands as co-catalysts toward Pd-catalyzed Heck–Mizoroki cross coupling reactions†

**DOI:** 10.1039/c9ra07912b

**Published:** 2019-11-21

**Authors:** Naima Munir, Sara Masood, Faroha Liaqat, Muhammad Nawaz Tahir, Sammer Yousuf, Saima Kalsoom, Ehsan Ullah Mughal, Sajjad Hussain Sumrra, Aneela Maalik, Muhammad Naveed Zafar

**Affiliations:** Department of Chemistry, Quaid-i-Azam University Islamabad-45320 Pakistan mnzafar@qau.edu.pk +923314503061; Department of Physics, University of Sargodha Sargodha-40100 Pakistan; H. E. J. Research Institute of Chemistry, International Centre for Chemical and Biological Sciences, University of Karachi 75270 Pakistan; Centre for Interdisciplinary Research in Basic Sciences (CIRBS), International Islamic University Islamabad Pakistan; Department of Chemistry, University of Gujrat Gujrat-50700 Pakistan; Department of Chemistry, COMSATS University Islamabad, Islamabad Campus Park Road Islamabad-45550 Pakistan aneela.maalik@comsats.edu.pk +923335490834

## Abstract

The present research work describes the synthesis of five new ligands containing pyridinium amine, [H_2_L^1^][OTf]_2_–[H_2_L^5^][I]_2_ from two new precursors, [P^3^_Et_][I] and [P^2^_Me_][CF_3_SO_3_]. The structure elucidations of the compounds were confirmed by multinuclear NMR (^1^H, ^13^C), FT-IR and by single crystal XRD techniques. Theoretical DFT studies were carried out to get better insight into the electronic levels and structural features of all the molecules. These synthesized new Pro-PYE ligands [H_2_L^1^][OTf]_2_–[H_2_L^5^][I]_2_ were found to be significantly active as co-catalysts for Pd(CH_3_CO_2_)_2_ toward Heck–Mizoroki coupling reactions with wide substrate scope in the order of [H_2_L^1^][OTf]_2_ ≫ [H_2_L^2^][OTf]_2_ > [H_2_L^3^][OTf]_2_ > [H_2_L^4^][OTf]_2_ > [H_2_L^5^][I]_2._

## Abstract 2

The present research work describes the synthesis of five new ligands containing pyridinium amine, [H_2_L^1^][OTf]_2_–[H_2_L^5^][I]_2_ from two new precursors, [P^3^_Et_][I] and [P^2^_Me_][CF_3_SO_3_].

## Graphical abstract

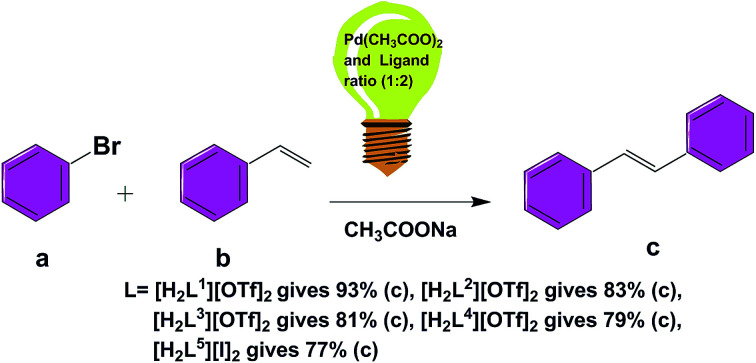

## 1. Introduction

**Fig. 1:**
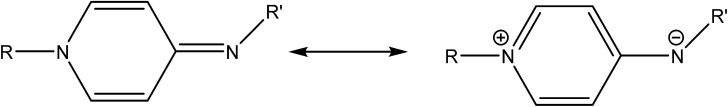
Possible resonance in PYE.

Electron donor ligands are essential for those metals undergoing various oxidative addition reactions. Phosphines and N-heterocyclic carbene (NHC) ligands are prevalent in this scenario owing to their remarkable electron donor characteristics.^1–3^ The steric hindrance of a substituted R group towards the metal in NHCs facilitates reductive elimination and product selectivity that mark this ligand as a superior choice in homogeneous catalysis, especially in cross-coupling chemistry.^4–7^ In depth study reveals that the tuning of the electronic density of a ligand is desirable according to the requirement of a coordinated metal to catalyze various stages of the redox process.^8,9^ Therefore the non-innocent ligands are ideally suited for this type of chemistry as they have the ability to switch and stabilize the various oxidation states of metals during chemical transformations.^10–12^ These ligands are promising in the field of catalysis as they are redox active spectator ligands. They not only block few coordination sites of the metal but also serve as electron reservoirs.^13,14^ Anionic ligand in resonance with cationic moiety can generate this kind of ligand. For example *N*-(1-methylpyridin-4(1*H*)-ylidene) amine (PYE) is a type of neutral nitrogen donor ligand that possesses electronic density adaptable to the requirement of metal owing to its resonance structures with varying charge on nitrogen from uni-negative to neutral (Fig. 1).^15^ The additional feature includes the exocyclic nitrogen atom of PYE that is pointed towards metal to control its steric environment.^16–18^

**Scheme 1:**
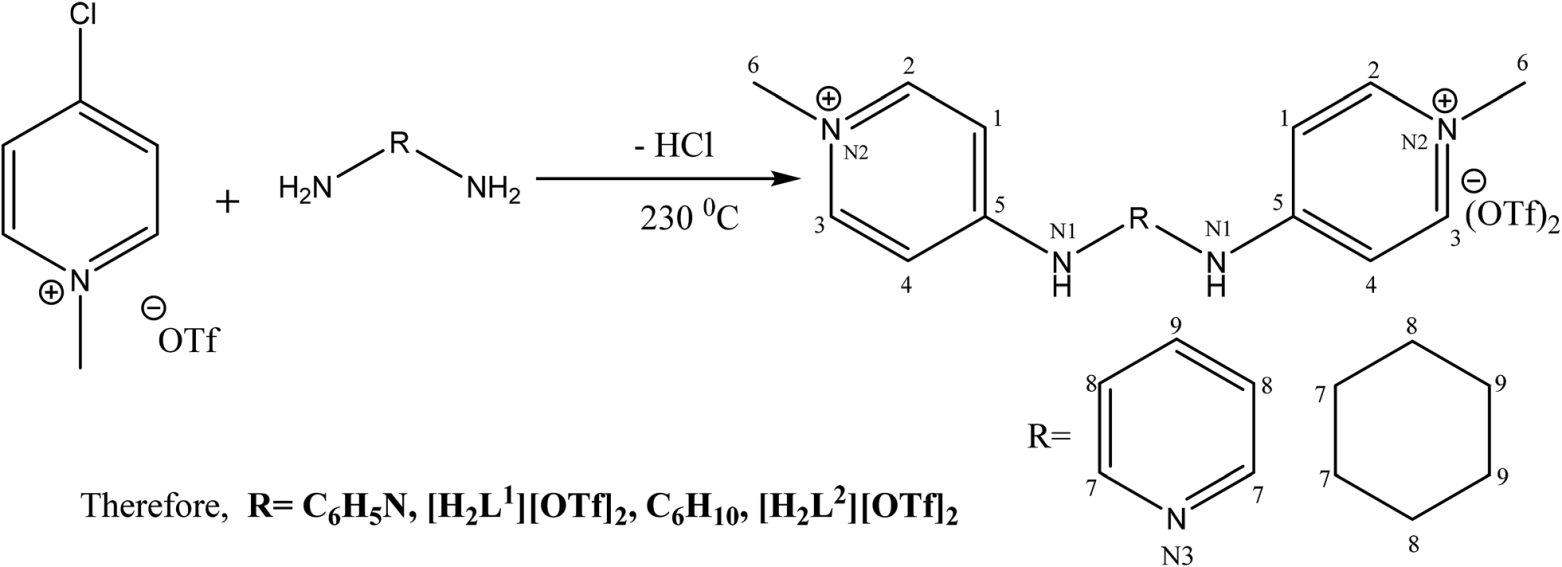
Synthesis of [H_2_L^1^][OTf]_2_ and [H_2_L^2^][OTf]_2_.

**Scheme 2:**
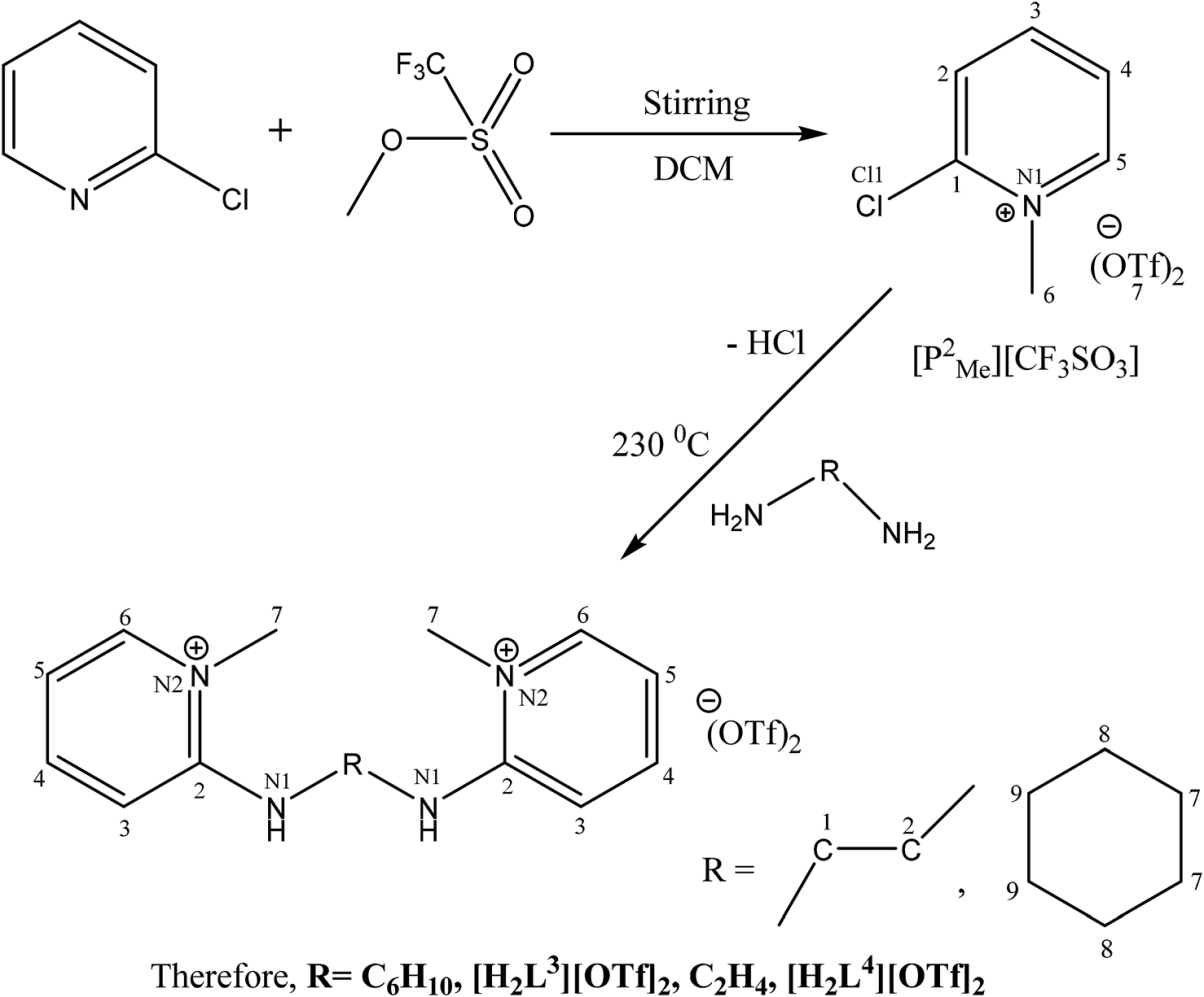
Synthesis of [H_2_L^3^][OTf]_2_ and [H_2_L^4^][OTf]_2_.

**Scheme 3:**
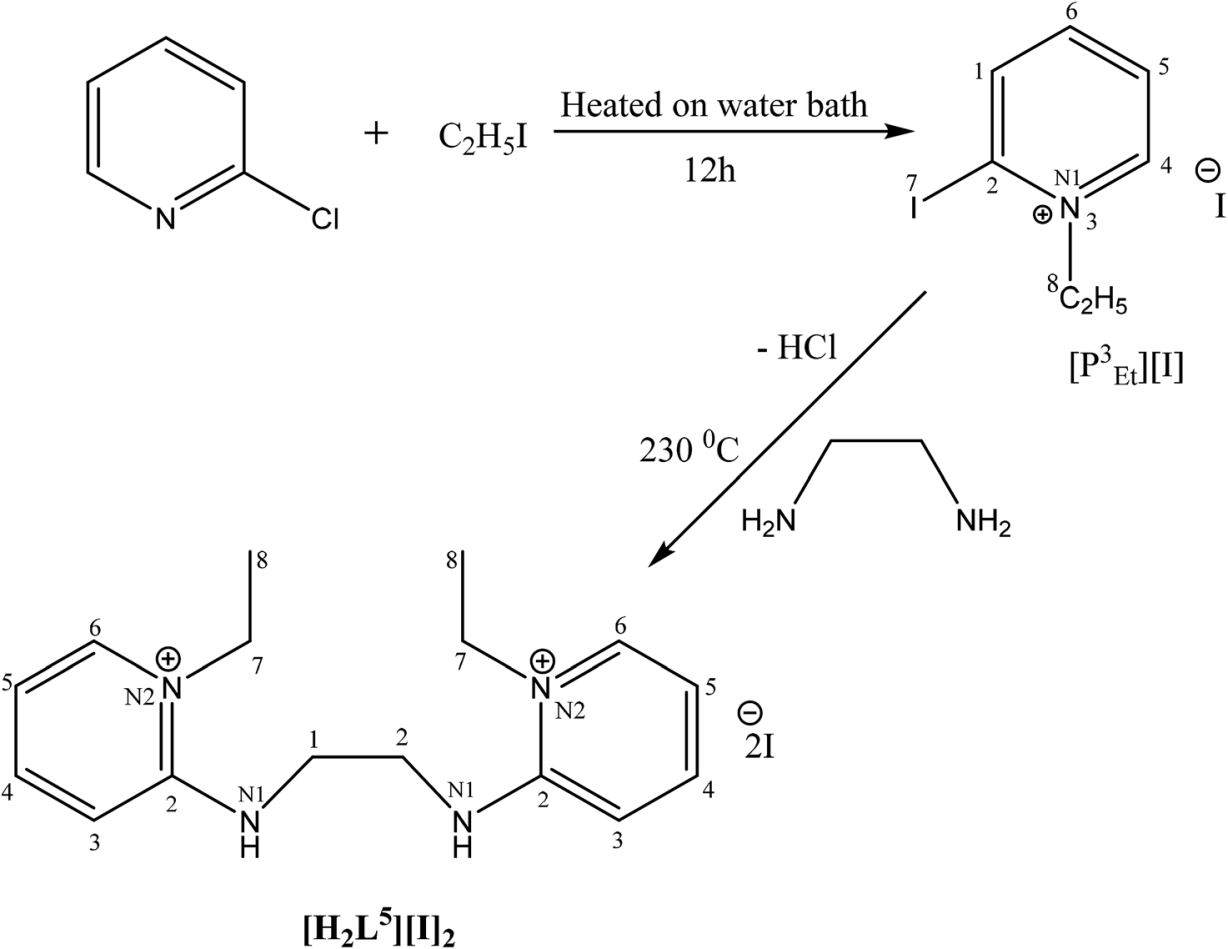
Synthesis of [H_2_L^5^][I]_2_.

The catalytic cycle of Heck–Mizoroki C–C cross coupling involves both oxidative addition as well as reductive elimination reaction. This coupling method is an appropriate synthetic route for synthesis of substituted olefins. High electronic density around the palladium *via* appropriate electron rich ligand facilitates oxidative addition (the key rate determining step) of various aliphatic or aromatic halides in the coupling reaction.^19–21^ Various palladium complexes have been used to catalyze above reaction.^22–24^ However, the much easier way is to use a mixture of suitable ligand and palladium precursor.^25,26^ In this regard, five new H-PYE ligands, [H_2_L^1^][OTf]_2_–[H_2_L^5^][I]_2_, were synthesized (Schemes 1–3) from two ligand precursors [P^3^_Et_][I] and [P^2^_Me_][CF_3_SO_3_]. These ligands were found to act as co-catalysts for Pd(OAc)_2_ and significantly enhanced their catalytic activity in Mizoroki–Heck coupling reactions under diverse reaction conditions. The molecular structures of all new compounds were characterized by using various spectroscopic and computational techniques.

## 2. Experimental

### 2.1. Reagents

Methyl triflate, 2-chloropyridine, cyclohexane-1,2-diamine, pyridine-2,6-diamine, ethane-1,2-diamine, aryl halides, ethyl acrylate and styrene purchased from Sigma Aldrich were used. Solvents were dried using standard procedures. *N*-Methyl-4-chloropyridinium triflate was synthesized by the reported method.^27^

### 2.2. Apparatus and instruments used

All reactions were performed under inert environment by Schlenk line technique. Melting point analysis was performed on Shimadzu melting point apparatus. Thermo Scientific spectrometer ranges from 4000–400 cm^−1^ was used to record FT-IR spectras. NMR spectra were recorded by using Bruker Avance Digital 300 MHz (^1^H) and 75 MHz (^13^C) at 300 K in DMSO-*d*_6_.

### 2.3. Chemistry

Syntheses of seven new compounds were shown in Schemes 1–3.

#### 2.3.1. Synthesis of [P^3^_Et_][I] (1)

2-Iodopyridinium ethyl iodide was prepared by treating 2-chloropyridine (4.91 mL, 53 mmol, 1 eq.) with ethyl iodide (12.78 mL, 159 mmol, 3eq.) in three neck round bottom flask (250 mL). Dichloromethane was also subjected to flask. The resulted mixture was subjected to heat under water bath for 12 hours. The product was cooled and washed by subsequent addition of acetone and pure product was achieved.

Royal yellow solid, yield 56%, mp: 156 °C. ^1^H NMR (300 MHz, DMSO-*d*_6_), *J* (Hz), *δ* (ppm): 9.28 (dd, 1H*, J* = 1.8, *Py-H*), 8.65 (dd, 1H, *J* = 1.8, *Py-H*), 8.19–8.07 (m, 2H, *Py-H*), 4.75 (q, 2H, *J* = 7.2, *CH*_*2*_), 1.49 (t, 3H, *J* = 7.2, *CH*_*3*_). ^13^C NMR (75 MHz, DMSO-*d*_6_), *J* (Hz), *δ* (ppm): 147.5, 144.7, 141.21, 127.87, 122.60, 62.31, 15.96. FT-IR (KBr, cm^−1^): 3069, 2956, 1600, 1357, 1272, 1153, 1065.

#### 2.3.2. Synthesis of [P^2^_Me_][CF_3_SO_3_] (2)

*N*-(1-Methylpyridin-2(1*H*)-ylidene) amine was synthesised by treating 2-chloropyridine (1 eq., 1.5 mL, 15.85 mmol) with methyl triflate (1.2 eq., 2.0 mL, 19.02 mmol) in DCM. After overnight stirring, the solvent was evaporated and product was precipitated out by addition of ether.

Colorless solid, yield 95%, mp: 161 °C. ^1^H NMR (300 MHz, DMSO-*d*_6_), *J* (Hz), *δ* (ppm): 9.16 (dd, 1H, *J* = 1.5, *Py-H*), 8.62–8.56 (m, 1H, *Py-H*), 8.37 (dd, 1H, *Py-H*), 8.12–8.07 (m, 1H, *Py-H*) 4.33 (s, 3H, *CH*_*3*_). ^13^C NMR (75 MHz, DMSO-*d*_6_), *J* (Hz), *δ* (ppm): 148.6, 147.4, 129.9, 126.5, 123.2, 119.0, 47.8. FT-IR (KBr, cm^−1^): 3097, 1617, 1309, 1260, 1142, 1028.

#### 2.3.3. Synthesis of [H_2_L^1^][OTf]_2_–[H_2_L^5^][I]_2_ (3–7)

##### [H_2_L^1^][CF_3_SO_3_]_2_ (3)

*N*-methylated-4-chloropyridinium triflate (0.5 g, 1.8 mmol, 2 eq.) and pyridine diamine (0.1 g, 0.9 mmol, 1 eq.) was subjected into two neck flask (100 mL). An argon atmosphere was provided to reaction mixture. The reaction was heated for about 3 hours at 230 °C. After the completion of reaction, solid was cooled to ambient temperature and scratched by spatula from flask. The off white crystals were dried *in vacuo* to give pure product. The crystals were grown in mixture of solvents as methanol and acetone.

Off white solid, yield 70%, mp: 165 °C. ^1^H NMR (300 MHz, DMSO-*d*_6_), *J* (Hz), *δ* (ppm): 11.06 (s, 2H, *NH*), 8.48–8.46 (d, 4H, *J* = 7.2, *py- H*), 7.96–7.91 (d, 1H, *J* = 7.7, *py-H*), 7.84–7.82 (d, 4H, *J* = 6.3, *py-H*), 6.96–6.94 (d, 2H, *J* = 8.1, *py-H*), 4.07 (s, 6H, *CH*_*3*_).^13^C NMR (75 MHz, DMSO-*d*_6_),*J* (Hz),*δ* (ppm): 152.6, 151.1, 145.1, 143.2, 112.9, 109.7, 45.8. FT-IR (KBR, cm^−1^): 3296, 3080, 1638, 1581, 1365, 1278, 1199, 1025.

##### [H_2_L^2^][CF_3_SO_3_]_2_ (4)

*N*-methylated-4-chloropyridinium triflate (1 g, 3.6 mmol, 2 eq.) along with cyclohexane diamine (0.21 mL, 1.8 mmol, 1 eq.) were added in two necked round bottom flask (50 mL). Under argon the temperature was provided to reaction mixture for about 150 minutes at 230 °C. The solid was cooled and recrystallised from methanol and acetone and dried *in vacuo* to give pure product [H_2_L^1^][OTf]_2_.

Brown crystalline solid, yield 78%, mp: 180 °C.^1^H NMR (300 MHz, DMSO-*d*_6_), *J* (Hz),*δ* (ppm): 9.11 (s, 2H, *NH*), 8.18–8.15 (d, 2H, *J* = 6.9, *py-H*), 7.93–7.91 (d, 2H, *J* = 6.9, *py-H*), 7.21–7.19 (m, 2H, *py-H*), 6.75–6.73 (m, 2H,*py-H*), 3.81 (s, 6H, CH_3_), 3.71–3.69 (d, 2H, *J* = 6, *cy-H*), 1.75–1.73 (d, 2H, *J* = 7.8, *cy-H*), 1.53–1.49 (d, 2H, *J* = 9.3, *cy-H*), 1.45–1.37 (m, 4H, *cy-H*).^13^C NMR (75 MHz, DMSO-*d*_6_), *J* (Hz),*δ* (ppm): 156.5, 144.3, 142.5, 110.4, 106.3, 56.2, 44.4, 31.5, 24.3. IR (KBR, cm^−1^): 3360, 3053, 2943, 1646, 1588, 1375, 1296, 1169, 1058.

##### [H_2_L^3^][CF_3_SO_3_]_2_ (5)

2-Chloropyridinium methyl triflate (0.5 g, 1.8 mmol, 2eq.) was added in a vacuum dried two neck round bottom flask (50 mL). Cyclohexane diamine (0.1 mL, 0.9 mmol 1 eq.) was introduced by glass syringe (100 μL) under argon into the flask. Then reaction mixture was heated for about 3 hours. Thermometer was used to monitor the temperature. After the completion of reaction, product was cooled to room temperature and scratched from the flask carefully *via* spatula. Desiccator was used to dry the solid. Good quality crystals can be grown in mixture of acetone and methanol.

Brown solid, yield 60%, mp: 232 °C. ^1^H NMR (300 MHz, DMSO-*d*_6_), *J* (Hz),*δ* (ppm): 8.26 (t, 2H, *J* = 6.6, *NH*), 8.24–8.18 (dd, 2H, *J* = 6.9, *py-H*), 7.99–7.37 (m, 2H, *py-H*), 7.03–7.00 (d, 1H, *J* = 7.5, *py-H*), 6.53–6.45 (dd, 1H, *J* = 8.4, *py-H*), 3.99 (s, CH_3_, 6H), 3.66 (s, 1H, *cy-H*), 3.54–3.50 (d, 1H, *J* = 3.6, *cy-H*), 2.49–2.25 (m, 2H, *cy-H*), 1.88–1.86 (d, 2H, *J* = 6 *cy-H*), 1.66–1.65 (d, 2H, *J* = 3, *cy-H*), 1.19–1.17 (d, 2H, *J* = 5.1, *cy-H*). ^13^C NMR (75 MHz, DMSO-*d*_6_), *J* (Hz), *δ* (ppm): 159.1, 153.3, 147.7, 143.2, 112.7, 43.2, 32.8, 24.5.

##### [H_2_L^4^][CF_3_SO_3_]_2_ (6)

Ethylene diamine (240.1 μL, 3.60 mmol) and *N*-methylated-2-chloropyridinium triflate (2 g, 7.2 mmol) were added in a 50 mL two neck round bottom flask under inert atmosphere and flask was heated for 2 hours at 180 °C. During this time, the solid reactant melts and undergoes melt reaction. After that time the melt was cooled to room temperature and the solid scratched from the flask. The solid was recrystallized from methanol to give pure dark brown crystalline product.

Dark brown solid, yield 70%; mp: 205 °C. ^1^H NMR (300 MHz, DMSO-*d*_6_), *J* (Hz),*δ* (ppm): 8.28 (t, 2H, *J* = 5.7, *NH*), 8.09–8.03 (m, 4H, *Py-H*), 7.42–7.39 (d, 4H, *J* = 9, *Py-H*), 7.05–6.99 (m, 2H, *Py-H*), 3.79 (d, 6H, *J* = 4.8, *CH*_*3*_), 3.67 (d, 4H, *J* = 4.5, *CH*_*2*_). ^13^C NMR (75 MHz, DMSO-*d*_6_), *J* (Hz),*δ* (ppm):153.2, 143.5, 142.4, 123.3, 118.9, 113.2, 111.5, 42.2, 40.7. IR (KBr, cm^−1^): 3325, 3086, 2952, 1649, 1591, 1348, 1243, 1159, 1025.

##### [H_2_L^5^][I]_2_ (7)

2-Iodo-1-ethyl-pyridinium iodide (0.2 g, 4.2 mmol, 2 eq.) was added in two necks round bottom flask. The flask was charged with ethylene diamine (0.138 mL, 2.07 mmol, 1 eq.) and potassium carbonate (K_2_CO_3_) (1.7 g, 12.46 mmol, 6 eq.) under inert argon. The mixture was stirred and refluxed in dichloromethane (DCM) for 20 hours at 80 °C. After refluxing, volatiles were removed. The product was recrystallized in DCM, collected and dried *in vacuo* to give pure product of orange yellow color.

Fawn yellow solid, yield 48%; mp: 304 °C. ^1^H NMR (300 MHz, DMSO-*d*_6_), *J* (Hz),*δ* (ppm): 8.28 (s, 1H, *N–H*), 8.19 (s, 1H, *Py-H*), 8.04 (s, 1H, *Py-H*), 7.40 (d, 1H, *Py-H*), 7.03 (s, 1H, *Py-H*), 4.22 (q, 2H, *J* = 5.1, *CH*_*2*_), 3.68 (s, 2H, *CH*_*2*_), 1.31 (t, 3H*, J* = 7.2, *CH*_*3*_). IR (KBr, cm^−1^): 3205, 3016, 2970, 1642, 1536, 1364, 1217, 1173, 1063.

#### 2.3.4. General method for the heck-coupling reaction

A dried evacuated tube (20 mL) fitted with Teflon tap was charged with Pd(OAc)_2_ (4.0 μmol, 0.01), ligand (4.0 μmol, 0.01), sodium acetate (1.1 eq., 440 μmol, 36 mg), magnetic stirrer under inert environment. Styrene (1.4 eq., 64 μL, 560 μmol), halobenzene (1.0 eq., 42 μL, 400 mmol) and dry DMA (2 mL) were added to the flask which was heated first at (130 °C) for 3 hours and then cooled to room temperature before use. On completion, the reaction flask was cooled at ambient temperature. After cooling, the mixture was extracted with ethyl acetate/n-hexane (1 : 5) solution. Column chromatography was employed to purify the product using ethyl acetate/n-hexane (20 : 80). Multinuclear NMR spectroscopy (^1^H NMR) confirmed the formation of product.

## 3. Results and discussion

### 3.1. Synthesis and characterization

The *N*-alkylation of 2-chloropyridine was carried out with ethyl iodide and methyl triflate to synthesize ligand precursors. Ethyl iodide being weak alkylating agent than methyl triflate required more vigorous conditions to yield [P^3^_Et_][I] as compared to [P^2^_Me_][CF_3_SO_3_]. During the synthesis of [P^3^_Et_][I], chloro attached at alpha carbon was substituted by iodo group and also appeared as counter anion, upon heating in water bath for 12 hours.^28,29^ The ethylation on pyridine was suggested by a quartet and triplet peaks at 4.75 and 1.49 ppm in proton NMR spectrum. The other ligand precursor, [P^2^_Me_][CF_3_SO_3_] was synthesized by just overnight stirring of 2-chloropyridine with methyl triflate. The methyl peak appeared at 4.33, 47.8 ppm in ^1^H and ^13^C NMR spectra respectively. The IR spectra of precursors [P^3^_Et_][I]–[P^2^_Me_][CF_3_SO_3_] showed aromatic stretch *ν*(C–H) at 3069–3085, aliphatic *ν*(C–H) at 2956, *ν*(C=N) at 1562–1588 cm^−1^, *ν*(C=C) at 1600–1646 cm^−1^, *ν*(C–N) at 1357–1303 cm^−1^, *ν*(C–I) at 445 cm^−1^, and *ν*(C–Cl) band at 633 cm^−1^ respectively. All remaining peaks appeared in their respective regions.

All the ligands, [H_2_L^1^][OTf]_2_–[H_2_L^5^][I]_2,_ were synthesised by the melt reaction between respective ligand precursor and corresponding amine. In these ligands, NH peak appeared in the range of 9–11 ppm that indicated the conversion of all primary amines to secondary amines. In aliphatic region, *N*-alkylated protons showed peaks for methyl and ethyl between 4.24–3.70 ppm in all ligands. Similarly carbon NMR showed these peaks between 62.3–47.8 ppm. The aromatic protons and their carbon atoms showed the respective peaks in their standard region. The peaks for cyclohexyl group appeared between 1.17–3.71 ppm for [H_2_L^2^][OTf]_2_ and [H_2_L^3^][OTf]_2_. In FT-IR spectra, the broad peaks appeared between 3296-3205 cm^−1^ for *ν*(N–H). While peaks of *δ*(N–H) appeared at 1581–1591 cm^−1^ in [H_2_L^1^][OTf]_2_–[H_2_L^5^][I]_2_. Peaks of aromatic region *ν*(C–H) ranges 3101–3016 cm^−1^ while skeleton vibrations or peaks due to breathing vibrations of aromatic ring were observed around the following frequencies; 1600, 1560, 1500, 1460 cm^−1^.^30^ In aliphatic region, the characteristic stretching band *ν*(C–H) appeared at 2970–2943 cm^−1^ and bending vibrations associated with *δ*(C–H) present in the particular range of 1436–1459 cm^−1^. Signals appeared for both secondary C=N and primary C–N stretch in the region of 1690–1590 cm^−1^ and 1350–1280 cm^−1^ respectively. Peaks between 1350–1225 cm^−1^ were assigned to both asymmetric and symmetric *ν*(S=O), while signals between 828–863 cm^−1^ were designated to *ν*(S–O). Sharp peak associated with *ν*(C–F) observed in the range of 1025–1063 cm^−1^.

**Fig. 2:**
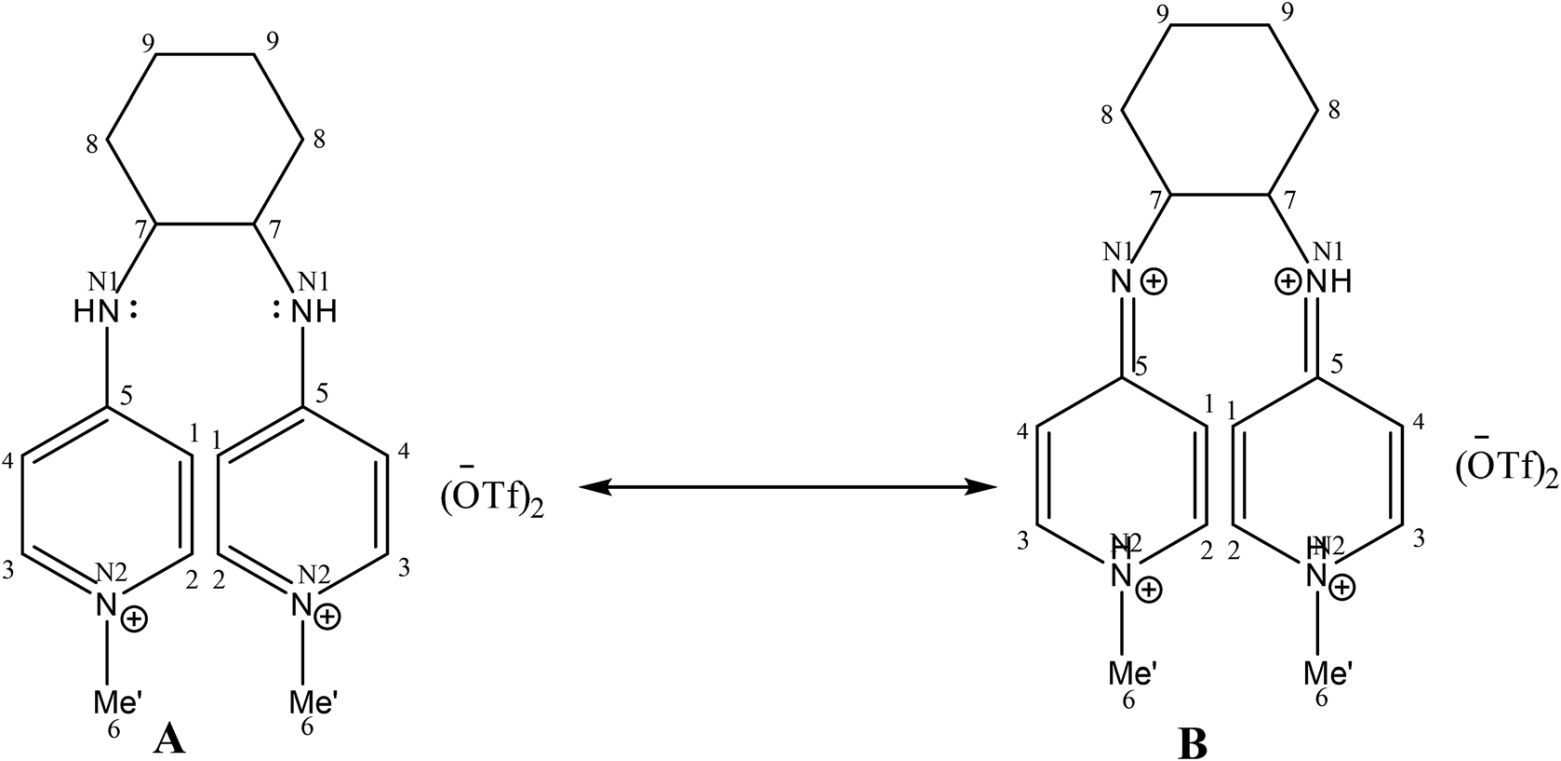
Resonance forms of [H_2_L^2^][OTf]_2_.

A comparison of proton and carbon NMR spectra of [H_2_L^1^][OTf]_2_ and [H_2_L^2^][OTf]_2_ reveals that B type resonance structure is predominant for [H_2_L^2^][OTf]_2_ unlike in [H_2_L^1^][OTf]_2_ as shown in Fig. 2. The ^1^H and ^13^C NMR (DMSO-*d*_6_) spectra for [H_2_L^2^][OTf]_2_ indicated the chemical equivalence in solution form of both pyridinium groups on NMR timescale. This lead to the appearance of only one signal for the both methyl groups at 3.81 ppm in ^1^H NMR and 44.4 ppm in ^13^C NMR spectra. Similarly, only one signal for NH appeared in ^1^H NMR spectrum at 9.11 ppm. Though the individual atoms making up each of the pairs C2/C3 and C1/C4 (as well as their symmetry related pairs) within each pyridinium group were found unequal and we observed separate signals in each case. Such as, for C2/C2, C3/C3, C1/C1 and C4/C4 in the ^13^C NMR spectrum, separate singlet signals were observed at 144.35, 142.46, 110.37, and 106.31 ppm, respectively. This situation most likely result because of the conformational changes that equilibrate the positions of each of the pairs of atoms, C2/C3, C1/C4, C2/C3 and C1/C4, were slow on the NMR timescale. Whereas the ^1^H and ^13^C NMR (DMSO-*d*_6_) spectra of [H_2_L^1^][OTf]_2_ showed the chemical equivalence of both pyridinium groups and the pairs of atoms C1/C4 and C2/C3 (and the corresponding pairs of atoms in the second pyridinium ring) in solution on NMR timescale. The interaction possibility of the N1 and N1 lone pairs in [H_2_L^1^][OTf]_2_ with the head pyridine ring resulting in reduction of the N1/C5 and N1/C5 π-interactions is one possible explanation for this different behavior. Hence rotation about the N1–C5 bond became rapid on the NMR timescale.

**Fig. 3:**
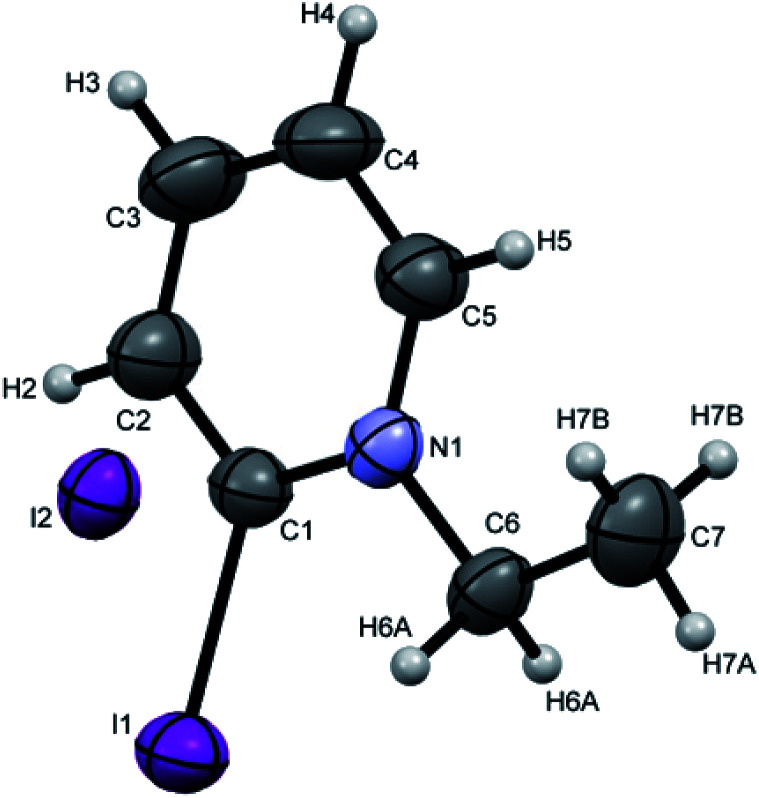
ORTEP depiction of [P^3^_Et_][I].

**Fig. 4:**
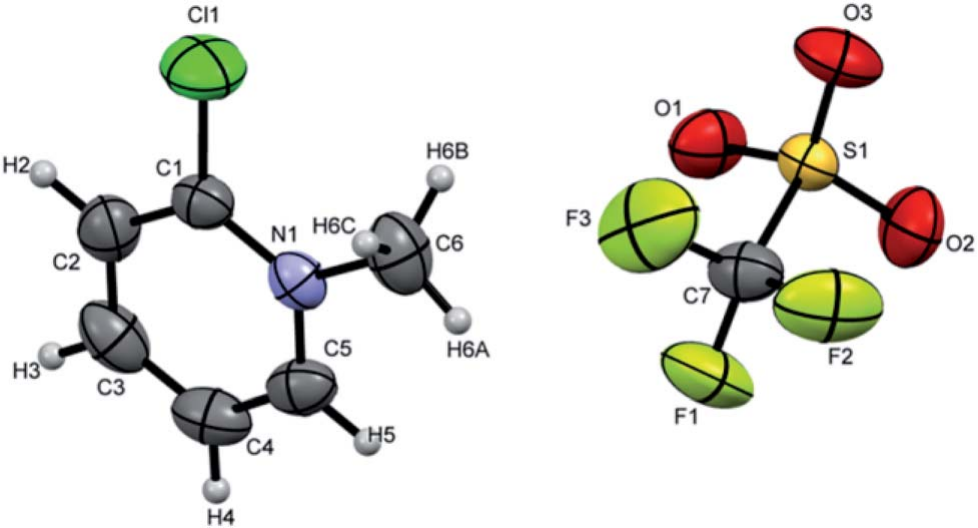
ORTEP depiction of [P^2^_Me_][CF_3_SO_3_].

Crystals suitable for single crystal XRD analysis were successfully grown for both precursors. [P^3^_Et_][I] crystals were grown from its acetone solution through slow evaporation method. The ORTEP depiction of [P^3^_Et_][I] is shown in Fig. 3. The crystal structure of [P^3^_Et_][I] confirms the successful ethylation of pyridine nitrogen. It also reveals that iodide acts as counter anion as well as replacement of *o*-chloro with iodo group. Crystals of [P^2^_Me_][CF_3_SO_3_] were prepared through slow evaporation process from its methanol solution. The ORTEP depiction of [P^2^_Me_][CF_3_SO_3_] is shown in Fig. 4 that shows successful methylation on nitrogen.

**Fig. 5:**
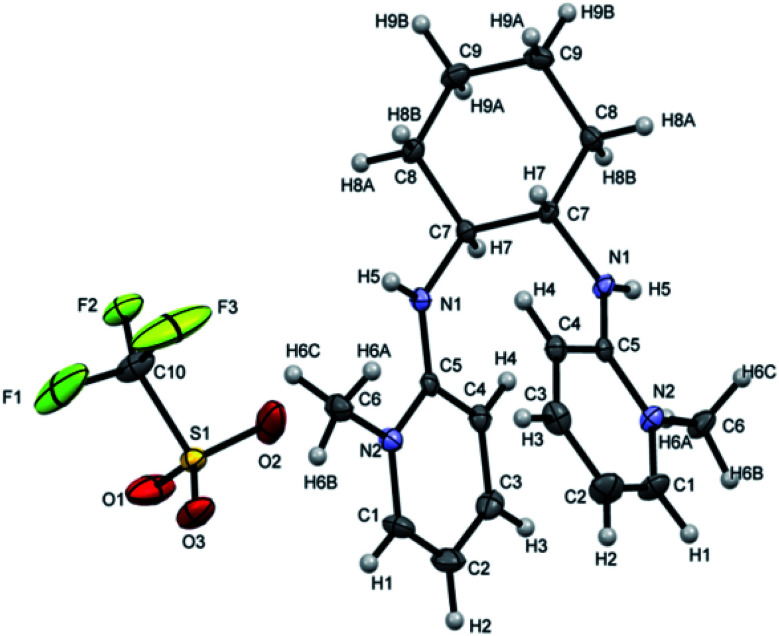
ORTEP depiction of [H_2_L^3^][OTf]_2_.

Attempts were made to grow the single crystals of all the ligands but [H_2_L^3^][OTf]_2_–[H_2_L^5^][I]_2_ were successfully grown and analyzed. [H_2_L^3^][OTf]_2_ was crystallized out in *Pbcn* space group from dichloromethane and ether (50 : 50) solution. ORTEP diagram is shown in Fig. 4. Crystal structure analysis confirms the attachment of two pyridinium rings to the cyclohexyl diamine and triflate is acting as counter anion. Cyclohexyl group adopts chair conformation with two substitutions at consecutive axial and equatorial positions show transoid geometry. Due to steric repulsion, the two substituted arms of cyclohexyl group shows torsion C7N1C5N2 angle of 171.8°.(Fig. 5).

**Fig. 6:**
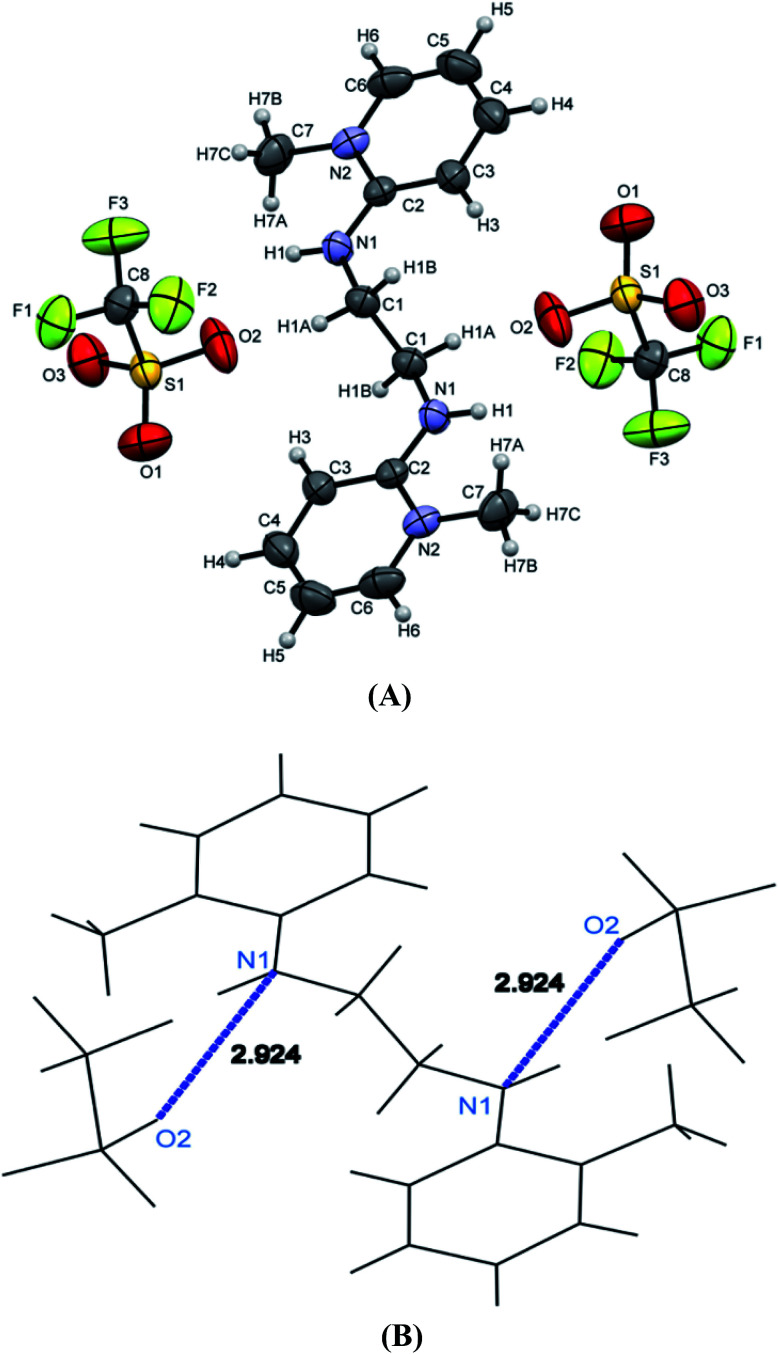
(A) ORTEP depiction of [H_2_L^4^][OTf]_2,_ (B) wire frame work of [H_2_L^4^][OTf]_2_ showing hydrogen bonding.

**Fig. 7:**
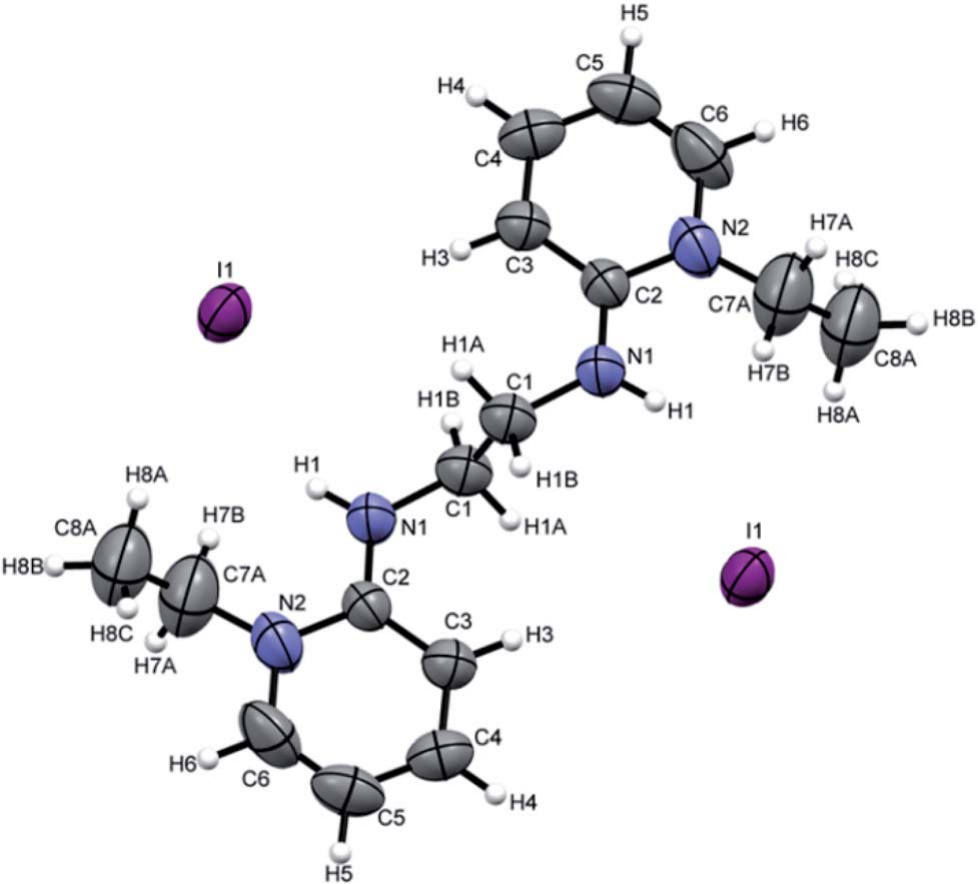
ORTEP depiction of [H_2_L^5^][I]_2_.

[H_2_L^4^][OTf]_2_ was crystallized out in the space group *P*2_1_/*c* in methanol and acetone solution. ORTEP depiction is shown in Fig. 6(A). Examination of crystal structure shows that there is torsion angle of 180.0° (N1–C1–C2–N3). Fig. 6(B) presents the hydrogen bonding that exists with NH–O distance of 2.92 Å (N1 and O2). These distances are within the normal range of NH–O bond lengths 2.81–3.04 Å.^31^. [H_2_L^5^][I]_2_ was crystallized out in the space group *R*3̄ in dichloromethane solvent. ORTEP depiction is shown in Fig. 7. Crystal structure cell coordinates reveal the presence of monoclinic system. The X-ray crystal structure confirms that two pyridinium rings are attached to nitrogen atoms of bidentate ethylene diamine. The two pyridinium amine groups beside ethylene unit adopt transoid geometry due to N1C1C1N1 torsion angle of precise 180° in both [H_2_L^4^][OTf]_2_ and [H_2_L^5^][I]_2_.

**Fig. 8:**
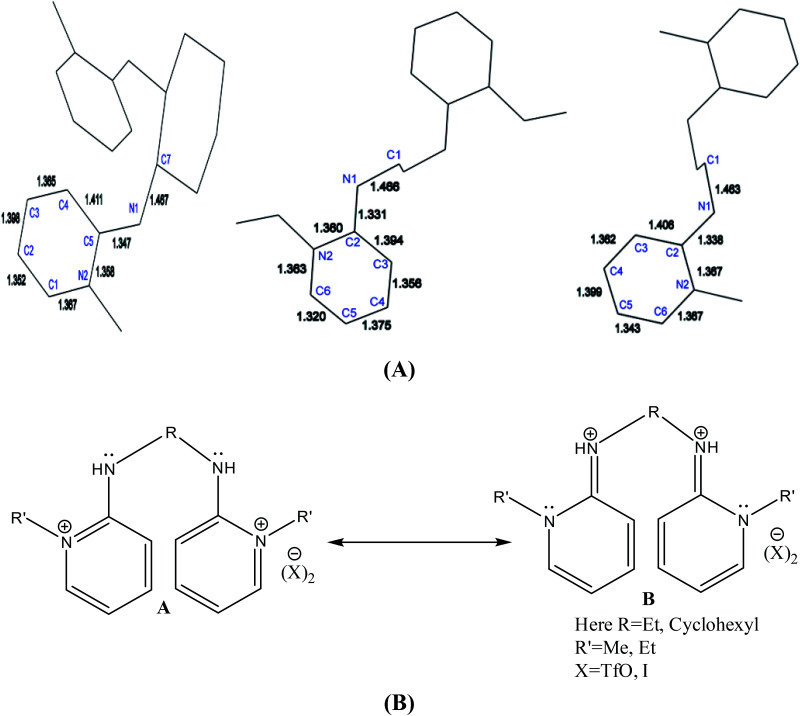
(A) Wire framework of [H_2_L^3^]^2+^–[H_2_L^5^]^2+^ showing important bond distances, (B) resonance forms of [H_2_L^3^][OTf]_2_–[H_2_L^5^][I]_2_.

The wire frame work structures of [H_2_L^3^]^+2^–[H_2_L^5^]^+2^ are shown in Fig. 8(A). It reveals that the bond lengths of [H_2_L^3^][OTf]_2_–[H_2_L^5^][I]_2_ are in accordance with the resonance form B in Fig. 8(B). The bond length of C5–N1 is 1.347 Å in [H_2_L^3^][OTf]_2._ Similarly [H_2_L^4^][OTf]_2_ and [H_2_L^5^][I]_2_ C2–N1 bond distances are 1.338 Å and 1.331 Å respectively that correspond to exocyclic carbon double bond nitrogen.

### 3.2. Computational analysis of molecular structures

Computational studies were carried out on all the compounds using Gaussian 09 software to gain a greater insight into the structural and electronic properties. The most significant edge to semi-empirical calculations is the prominent increase in computational accuracy without further increase in computing time. The geometry optimization of the structures was performed using AM1 semi-empirical method and an energy calculation was carried out for each optimized structure to find the energy minimum, (in the presence and absence of ligands). The energy of the frontier molecular orbitals (HOMO–LUMO) was also calculated (and confirmed to earlier results by Gaussian) by MOE 2016 software^32^ using semi-empirical method with a Hamiltonian force field, MMFF94x and 0.0001 Gradient. All the computed data available in this study shows highly convergent values obtained from energy minimization of compounds using Gaussian and MOE.

A number of structural parameters have been obtained from semi-empirical studies on conformers with minimum energy. The energy of the frontier molecular orbitals is also instrumental in obtaining the values of chemical hardness (*η*), chemical potential (*χ*) and electrophilicity index (*ω*) using the eqn (1)–(3);^33,34^ where *I* and *A* are the ionization potential and electron affinity of a molecule, shown in Tables 1 and 2.

1 *η* = (*I* − *A*)/2

2 *χ* = −(*I* + *A*)/2

3 *ω* = *χ*^2^/2*η*

**Table 1:** Ionization energy, electron affinity and band gap values of [P^3^_Et_][I]–[H_2_L^5^][I]_2_

Ligands	Values (eV)	Ionization energies (eV per molecule)	Electron affinity (eV per molecule)	Band gap (eV per molecule)
[P^3^_Et_][I]	HOMO = −8.15, LUMO = −0.47	−8.15	−0.47	−7.67
[P^2^_Me_][CF_3_SO_3_]	HOMO = −9.39, LUMO = −2.27	−9.39	−2.27	−7.12
[H_2_L^1^][OTf]_2_	HOMO = −14.69, LUMO = −6.77	−14.69	−6.77	−8.22
[H_2_L^2^][OTf]_2_	HOMO = −14.50, LUMO = −6.12	−14.50	−6.12	−8.38
[H_2_L^3^][OTf]_2_	HOMO = −9.49, LUMO = −2.55	−9.49	−2.55	−6.94
[H_2_L^4^][OTf]_2_	HOMO = −9.15, LUMO = −2.33	−9.15	−2.33	−6.82
[H_2_L^5^][I]_2_	HOMO = −14.82, LUMO = −6.75	−14.82	−6.75	−8.07

**Table 2:** Chemical hardness (*η*), chemical potential (*χ*) and electrophilicity index (*ω*) of [P^3^_Et_][I]–[H_2_L^5^][I]_2_

Ligands	Chemical hardness (*η*)	Chemical potential (*χ*)	Electrophilicity index (*ω*)
[P^3^_Et_][I]	−4.31	4.31	−2.15
[P^2^_Me_][CF_3_SO_3_]	−5.83	5.83	−2.91
[H_2_L^1^][OTf]_2_	−10.73	10.73	−5.36
[H_2_L^2^][OTf]_2_	−10.31	10.31	−5.15
[H_2_L^3^][OTf]_2_	−6.02	6.02	−3.01
[H_2_L^4^][OTf]_2_	−5.74	5.74	−2.87
[H_2_L^5^][I]_2_	−10.78	10.78	−5.39

**Fig. 9:**
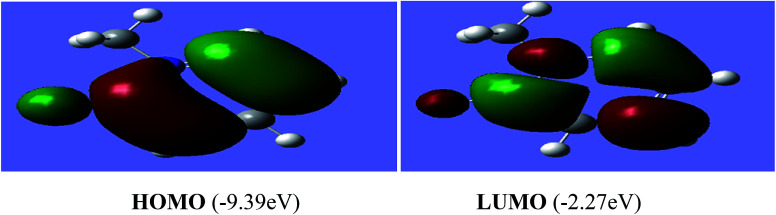
Electron density distribution of HOMO and LUMO of [P^3^_Et_][I].

**Fig. 10:**
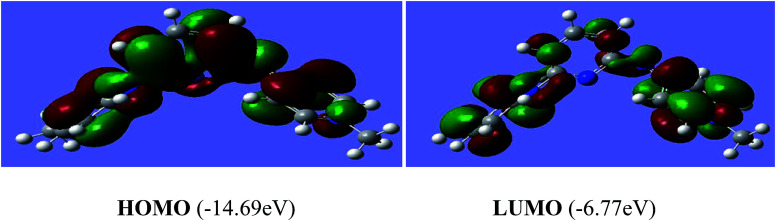
Electron density distribution of HOMO and LUMO of [H_2_L^1^][OTf]_2_.

The reactivity of a compound can also be gauged from the HOMO and LUMO orbitals energy difference (Δ*E*_H−L_); a larger band gap indicates lower reactivity and higher chemical hardness.^35,36^ The greater is the energy of HOMO, greater would be ionization potential and thus more is the ability of a molecule to donate an electron pair. While LUMO has the ability to accept electron density *via* back bonding from transition metal that is associated with electron affinity of molecule.^37,38^ The pictorial representations of all the frontier molecular orbitals (HOMO–LUMO) were acquired. HOMO–LUMO of [P^3^_Et_][I] and [H_2_L^1^][OTf]_2_ are shown in Fig. 9 and 10. The important bond lengths of optimized structures and XRD structures of compounds [H_2_L^3^][OTf]_2_–[H_2_L^5^][I]_2_ are compared in Tables 3–5. It shows good agreement between the computed and experimental values for all the compounds. Slight divergences in some bond lengths can be attributed to the choice of computational method, and the basis set used.^35–39^

**Table 3:** Experimental and theoretical bond lengths [Å] of [H_2_L^3^][OTf]_2_

Concerned atoms	Experimental	Calculated	Concerned atoms	Experimental	Calculated
C1–C2	1.35	1.38	N2–C1	1.36	1.35
C2–C3	1.39	1.38	C5–N1	1.35	1.39
C3–C4	1.36	1.39	N1–C7	1.46	1.46
C4–C5	1.41	1.41	C7–C8	1.53	1.54
C5–N2	1.36	1.37	C8–C9	1.53	1.53
N2–C6	1.47	1.48	C7–C7	1.54	1.55

**Table 4:** Experimental and theoretical bond lengths [Å] of [H_2_L^4^][OTf]_2_

Atom no	Experimental	Calculated	Atom no	Experimental	Calculated
C1–N1	1.46	1.48	C5–C6	1.34	1.38
N1–C2	1.34	1.34	C6–N2	1.36	1.35
C2–C3	1.40	1.41	N2–C7	1.46	1.48
C3–C4	1.36	1.39	N2–C2	1.36	1.38
C4–C5	1.39	1.38	C1–C1	1.51	1.52

**Table 5:** Experimental and theoretical bond lengths [Å] of [H_2_L^5^][I]_2_

Concerned atoms	Experimental	Calculated	Concerned atoms	Experimental	Calculated
C1–N1	1.46	1.45	C5–C6	1.32	1.38
N1–C2	1.33	1.39	C6–N2	1.36	1.36
C2–C3	1.39	1.41	N2–C7	1.49	1.49
C3–C4	1.36	1.39	C7–C8	1.46	1.52
C4–C5	1.37	1.38	C5–C6	1.32	1.38

### 3.3. Evaluation of Mizoroki–Heck cross-coupling reaction

The estimation of potential efficiency of [H_2_L^1^][OTf]_2_–[H_2_L^5^][I]_2_ as a co-catalysts toward Heck coupling reaction were evaluated in the presence of palladium acetates catalyst. The results obtained are tabulated in Table 6. [H_2_L^1^][OTf]_2_ was used for the optimization of various parameters such as amount of catalyst loading (Table 6, entry 1–5), solvent system (Table 6, entry 5–9), base (Table 6, entry 10–15), temperature (Table 6, entry 15–19) and molar ratio of palladium acetate to ligand (Table 6, entry 19–20, 21, 22), with pure Pd (OAc)_2_ catalyst and without any ligand reaction time 4 hours resulted in lowest 6% yield (Table 6, entry 20). Initially, bromobenzene with styrene reaction was selected for optimization of reaction conditions. A reaction with only palladium acetate or ligand yield trace amount of product (Table 6, entry 1 or 20). The optimum reaction conditions were obtained for (entry 19, Table 6); 0.01 mol% palladium acetate, DMF solvent, NaOAc 1.1 mmol, 130 °C and 1 : 1 palladium acetate to [H_2_L^1^][OTf]_2_.

**Table 6:** Optimization of catalytic Mizoroki–Heck cross coupling reaction^a^

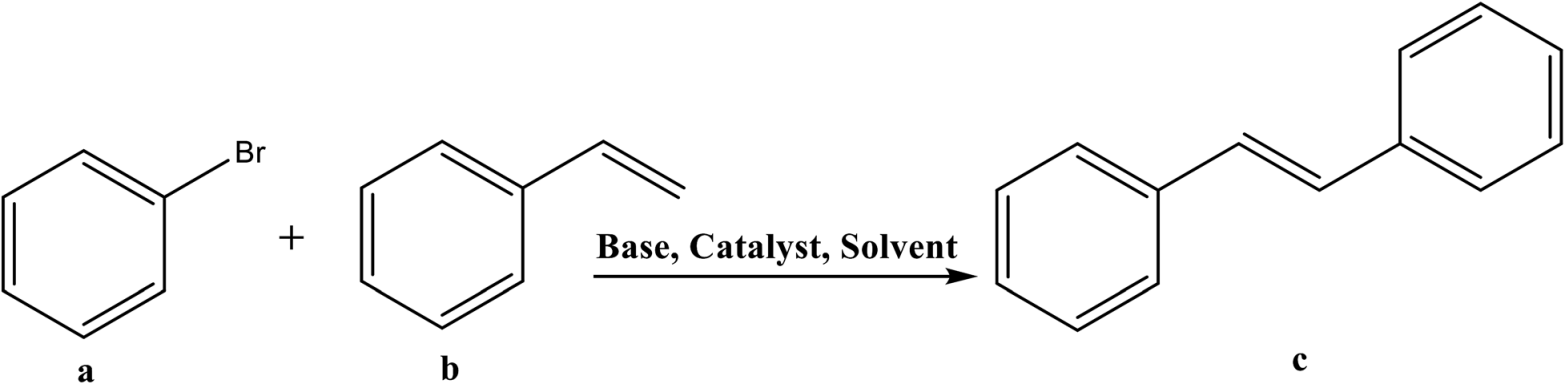						
Entry	Base	Pd(OAc)_2_ (mol)	Pd(OAc)_2_ : L	Temperature (°C)	Solvent	Yield (%)
1	K_2_CO_3_	—	0 : 1 without catalyst	100	DMA	No reaction
2	K_2_CO_3_	0.1	1 : 1	100	DMA	83
3	K_2_CO_3_	0.05	1 : 1	100	DMA	80
4	K_2_CO_3_	0.01	1 : 1	100	DMA	78
5	K_2_CO_3_	0.001	1 : 1	100	DMA	50
6	K_2_CO_3_	0.01	1 : 1	80	Hexane	43
7	K_2_CO_3_	0.01	1 : 1	110	Toluene	47
8	K_2_CO_3_	0.01	1 : 1	100	H_2_O	56
9	K_2_CO_3_	0.01	1 : 1	75	DMF	68
10	K_2_CO_3_	0.01	1 : 1	100	DMF	84
11	Na_2_CO_3_	0.01	1 : 1	100	DMF	80
12	Cs_2_CO_3_	0.01	1 : 1	100	DMF	78
13	NEt_3_	0.01	1 : 1	100	DMF	41
14	Pyridine	0.01	1 : 1	100	DMF	46
15	CH_3_COONa	0.01	1 : 1	100	DMF	86
16	CH_3_COONa	0.01	1 : 1	25	DMF	53
17	CH_3_COONa	0.01	1 : 1	50	DMF	63
18	CH_3_COONa	0.01	1 : 1	75	DMF	72
19	CH_3_COONa	0.01	1 : 1	130	DMF	93
20	CH_3_COONa	0.01	1 : 0 without ligand	130	DMF	6
21	CH_3_COONa	0.01	1 : 2	130	DMF	54
22	CH_3_COONa	0.01	2 : 1	130	DMF	56

a Conditions: 3 h except entry 20, styrene (1.4 eq.) *versus* halobenzene. Isolated yields were based on aryl halide.

**Fig. 11:**
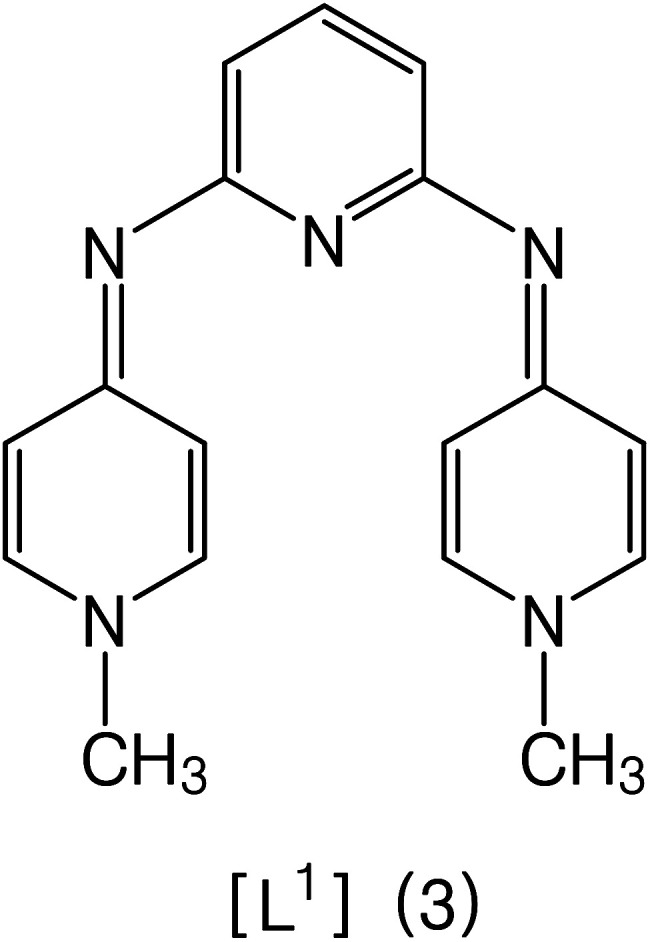
Proposed structure of L^1^.

All the other synthesized ligands, [H_2_L^2^][OTf]_2_–[H_2_L^5^][I]_2_, were compared with [H_2_L^1^][OTf]_2_ and the results are shown (Table 7). [H_2_L^1^][OTf]_2_ presented maximum coupled product comparatively. The reason can be associated with the extensive delocalization of electrons in the *in situ* generated L^1^ (Fig. 11) that binds with palladium acetate. This conjugation in ligand can make amido nitrogen electron rich that in turn increases the electronic density on palladium catalyst that eventually speeds up the rate of oxidative addition of bromobenzene, thus activating the catalyst towards coupling reaction.

**Table 7:** Comparison of [H_2_L^1^][OTf]_2_–[H_2_L^5^][I]_2_ for Mizoroki–Heck cross coupling reactions^a^

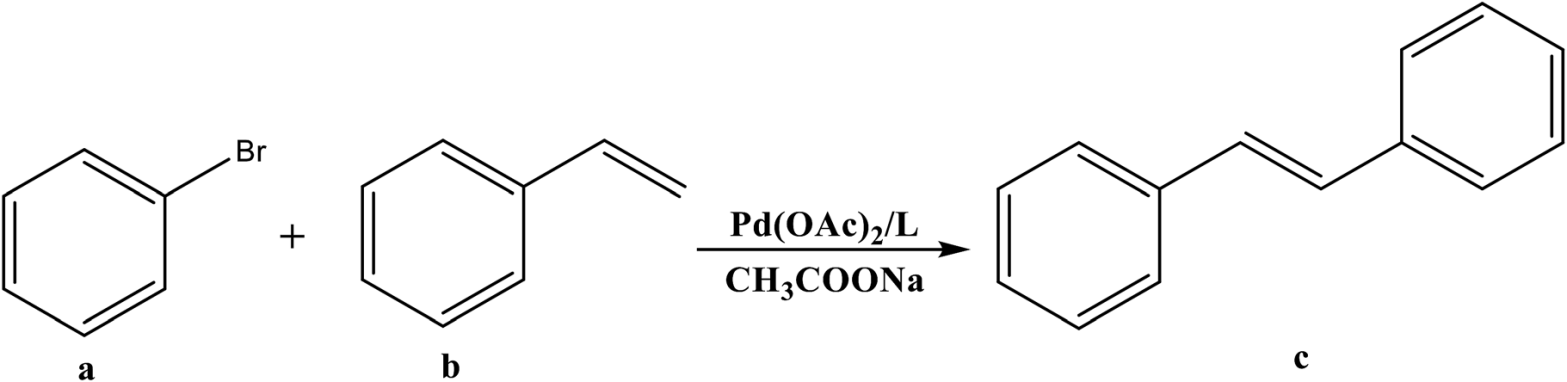		
Entry	Ligands	%Yield
1	[H_2_L^1^][TfO]_2_	93
2	[H_2_L^2^][TfO]_2_	83
3	[H_2_L^3^][TfO]_2_	81
4	[H_2_L^4^][TfO]_2_	79
5	[H_2_L^5^][I]_2_	77

a Conditions: 3 h, 130 °C, styrene (1.4 eq.) *versus* halobenzene, Pd(OAc)_2_ : L = 1 : 1, DMF (2 mL), ligand loading (0.01 mol), Pd(OAc)_2_ loading (0.01 mol) and NaOAc (4.3 mmol, 3.1 eq.). Isolated yield based on aryl halide.

Alkyl substituted ligands, [H_2_L^2^][OTf]_2_–[H_2_L^5^][I]_2_, were found less active comparatively. Nevertheless, they showed coupled product in the range of 77 to 83% owing to the inductive effect of ethyl and hexyl groups. The minor variations in product yield can be related to the steric of *N*-alkylated group of varying degree around the donor amido nitrogen. This catalytic activity order of co-catalysts can also be related with their *E*_HOMO_ values *i.e.*, greater is the energy of HOMO, greater would be the electron donation from ligand to Pd(ii) thus more would be the stability of *in situ* generated active catalyst.

Substrates with a different range of electron withdrawing to electron donating substituent were coupled under optimum conditions to test the substrate scope of best catalyst system ([H_2_L^1^][OTf]_2_/Palladium acetate). As expected, more activated aryl iodides couple more effectively than aryl bromides and aryl chloride (Table 8, entry 1–8). The high reactivity of aryl iodides are due to small dissociation energy of C–I bond and ease of activation towards Pd(0) catalyst in oxidative addition step.^40^ Generally coupling of aryl chlorides demand high catalyst loading with low yield of coupled product.^41^

**Table 8:** Synthesis of aryl halides catalyzed by Pd(OAc)_2_/L through Mizoroki–Heck cross-coupling reaction^a^

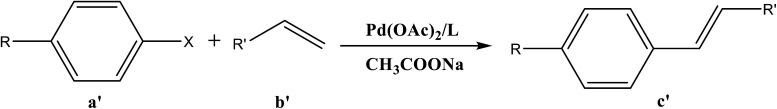				
Entry	RC_6_H_4_X	R'CHCH_2_	Yield (%)	Time (h)
1	ClC_6_H_5_	C_6_H_5_CHCH_2_	67	6
2	4-ClC_6_H_4_CH_3_	C_6_H_5_CHCH_2_	65	7
3	4-ClC_6_H_4_NO_2_	C_6_H_5_CHCH_2_	72	6
4	4-ClC_6_H_4_COCH_3_	C_6_H_5_CHCH_2_	70	6
5	I–C_6_H_5_	C_6_H_5_CHCH_2_	98	4
6	4-IC_6_H_4_CH_3_	C_6_H_5_CHCH_2_	95	5
7	BrC_6_H_5_	C_6_H_5_CHCH_2_	93	4
8	4-BrC_6_H_4_CH_3_	C_6_H_5_CHCH_2_	89	6
9	4-BrC_6_H_4_OCH_3_	C_6_H_5_CHCH_2_	87	6
10	4-BrC_6_H_4_NO_2_	C_6_H_5_CHCH_2_	96	4.5
11	4-BrC_6_H_4_CHO	C_6_H_5_CHCH_2_	92	4
12	4-BrC_6_H_4_COCH_3_	C_6_H_5_CHCH_2_	94	4
13	1-BrC_10_H_7_	C_6_H_5_CHCH_2_	85	16
14	BrC_6_H_5_	CH_2_CHCO_2_C_2_H_5_	96	3.5
15	4-BrC_6_H_4_CH_3_	CH_2_CHCO_2_C_2_H_5_	94	4
16	4-BrC_6_H_4_OCH_3_	CH_2_CHCO_2_C_2_H_5_	93	4
17	4-BrC_6_H_4_NO_2_	CH_2_CHCO_2_C_2_H_5_	98	3
18	4-BrC_6_H_4_CHO	CH_2_CHCO_2_C_2_H_5_	97	2
19	4-BrC_6_H_4_COCH_3_	CH_2_CHCO_2_C_2_H_5_	98	2
20	1-BrC_10_H_7_	CH_2_CHCO_2_C_2_H_5_	92	12

a Conditions: 130 °C, styrene (1.4 eq.) *versus* halobenzene, Pd(OAc)_2_ : L = 1 : 1, NaOAc (4.3 mmol, 3.1 eq.), DMF (2 mL), ligand loading 0.01, Pd(OAc)_2_ loading (0.01 mol%). Isolated yield based on aryl halide.

Likewise, those aryl halides with electron donating substituents like methoxy, methyl or naphthyl and electron neutral group such as –H showed less reactivity than aryl halide containing electron withdrawing substituents such as nitro, aldo and keto groups (Table 8, entry 1–13). The more electron deficient quaternary carbon of aryl halide better would be the rate of oxidative addition reaction. Similar trend was shown by ethyl acrylate for the above mentioned aryl halides activated to different degrees. However the yield of all coupled products with ethyl acrylate was comparatively more than styrene (Table 8, entry 14–18). The reason can be attributed to the greater polarization of the alkene with more electron-withdrawing adjacent carbonyl group in ethyl acrylate. This polarization enhances the insertion of ethyl acrylate into aryl–palladium bond during catalytic cycle. Moreover, as explained earlier, out of 3.1 eq. sodium acetate, 1.1 eq. of sodium acetate was used for catalytic reaction however remaining 2 eq. of sodium acetate was used to *in situ* deprotonate the ligand leading to the formation of an effective complex with ligand.

Efforts to synthesize pure palladium complexes with all the ligands, [H_2_L^1^][OTf]_2_–[H_2_L^5^][I]_2_ were proved to be futile owing to strong trans influence by *in situ* generated deprotonated ligands. The resulted mixtures were attempted to be purified by various analytical techniques but was proved in vain. To full fill the urge of identifying the actual nature of catalyst, standard mercury test was performed. 150 to 300 equivalent of Hg(0) compared to catalytic system was added in the catalytic reactor during coupling reaction. The yield of coupled product was not affected in this test reaction. This result clearly confirmed the *in situ* generation of homogeneous palladium catalysts and heterogeneous Pd(0) catalysts were not obtained by any opponent way.^42,43^

**Fig. 12:**
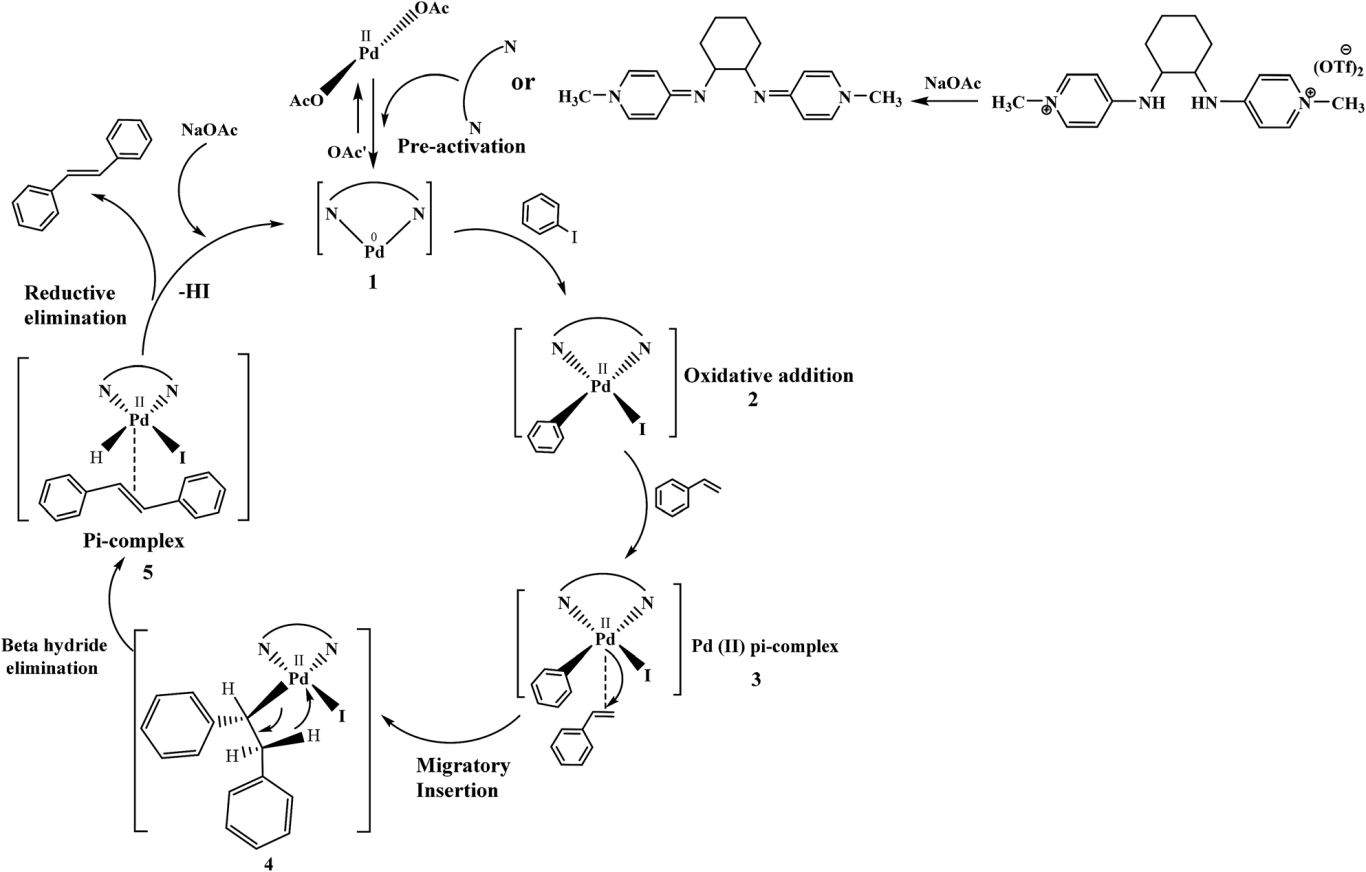
The catalytic cycle showing the role of reagents, ligands and *etc.*, under the best reaction conditions.

The plausible catalytic cycle of one of the reactions between iodobenzene and styrene under the best reaction conditions (mentioned in Table 8) shown in Fig. 12 involved pre-activation of catalyst followed by oxidative addition of palladium in benzene to iodide bond of iodobenzene, Pd(ii) π-complex formation, migratory insertion of styrene in palladium and carbon bond, relief of torsional strain with the elimination of beta hydride, another π-complex formation with stilbene and generation of Pd(0) catalyst with reductive elimination. Sodium acetate plays a vital role in regeneration of catalyst.^44^

Herein, we performed the critical comparison (mentioned in Table 9) of the catalytic activity (entry 1 to 5)^45*a*–*e*^ of this work in entry 6 related to Heck coupling of chlorobenzene and styrene with literature showed that this catalyst is potentially the trademark for the coupling of chlorobenzene with styrene. It gave 67% yield in 6 hours at 130 °C with 1 mol%. The problem with catalysts in entries 1 and 2 catalysts is utilization of too much time for catalytic conversion of chlorobenzene to coupled product. The catalyst in entry 3 yields 70% coupled product in just 85 minutes at 120 °C but with much high catalyst amount *i.e.* 10 mol%. The best one seems to be in entry 4; utilizes less catalyst at the cost of 20 °C more temperature comparatively. The catalyst in entry 5 is useless for chlorobenzenes. Whereas with respect to catalyst shown in entry 4, our catalysts showed more substrate scope for chlorobenzenes.

**Table 9:** Comparison of developed catalytic system (entries 6, 14, 16) with the literature reported systems (entries 1–5, 7–11, 13–15) showing different ligands and conditions^a^

Entry	Reactions	Halo-benzene (mmol)	Ligands (L), Pd(OAc)_2_ mol%	Solvent (ml), base	Time (h)	*T* (°C)	*Y* (%)	Ref.
1	C_6_H_5_Cl + styrene	1	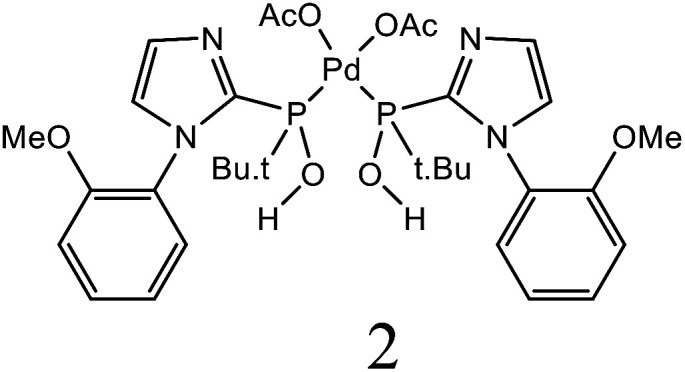	DMF, K_2_CO_3_	12	60	62	45*a*
2	C_6_H_5_Cl + styrene	0.5	Pd(OAc)_2_: Dave-Phos (2 : 6) 2	1,4- dioxane, TBAE	24	80	99	45*b*
			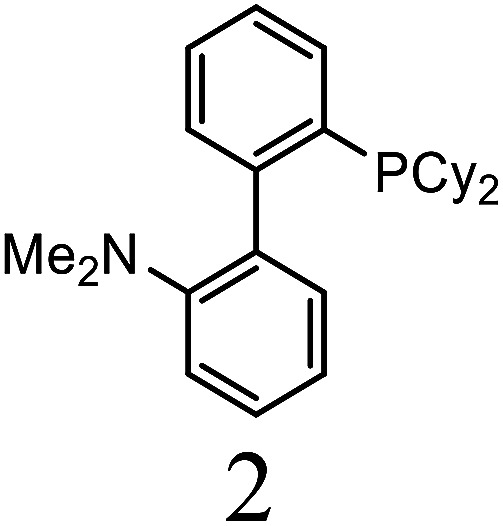					
3	C_6_H_5_Cl + styrene	1	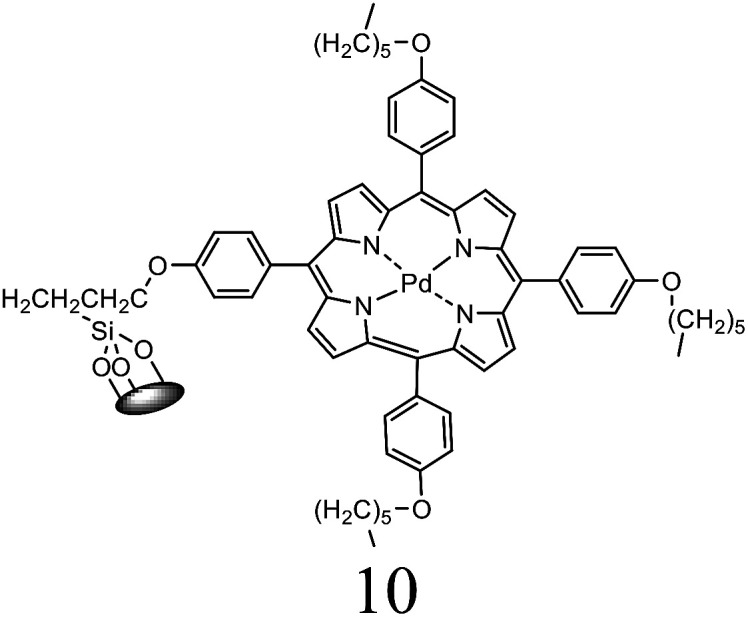	K_2_CO_3_, DMF	85 min	120	70	45*c*
4	C_6_H_5_Cl + styrene	1.5	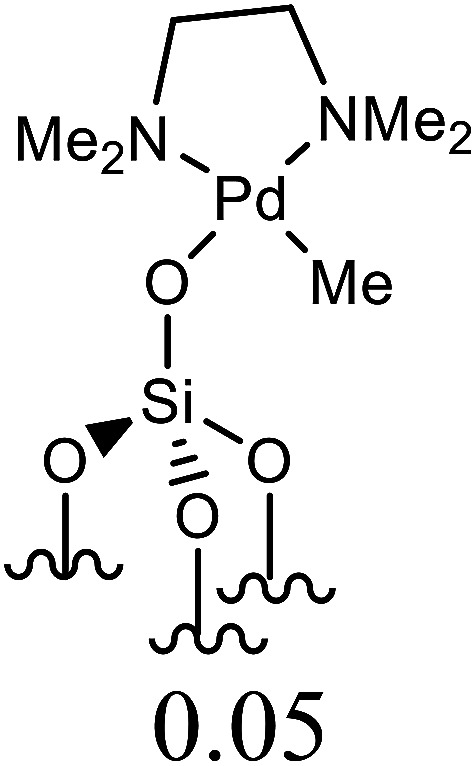	NMP, Ca(OH)_2_	6	150	62	45*d*
5	C_6_H_5_Cl + styrene	1	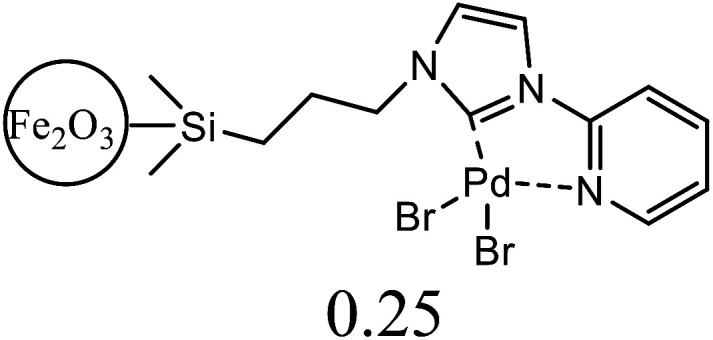	NMP, K_2_CO_3_	2.5	120	Trace	45*e*
6	C_6_H_5_Cl + styrene	400	Pd (OAc)_2_ : [H_2_L^1^][TfO]_2_ (1 : 1)	DMF, NaOAc	6	130	67	This work
7	C_6_H_5_I + styrene	1	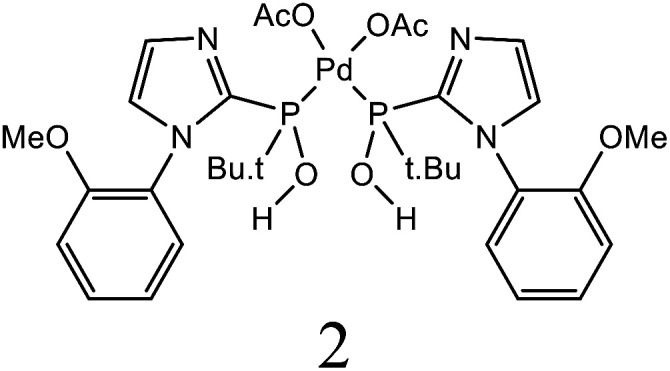	DMF, K_2_CO_3_	12	60	98	45*a*
8	C_6_H_5_I + styrene	1	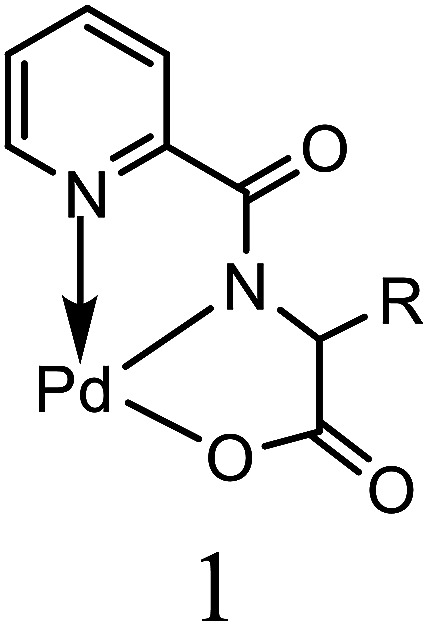	DMF, Na_2_CO_3_	20	145	95	45*f*
9	C_6_H_5_I + styrene	1	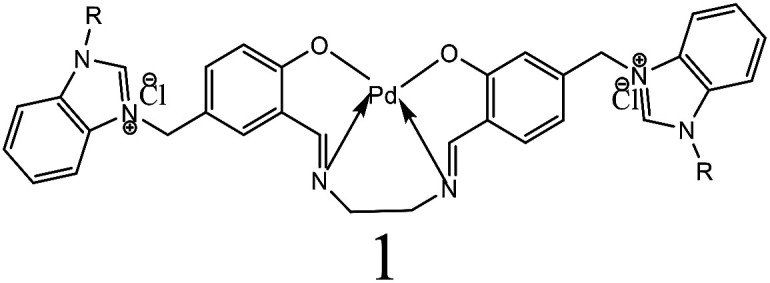	DMF, K_2_CO_3_	20	130	90	45*g*
10	C_6_H_5_I + styrene	1	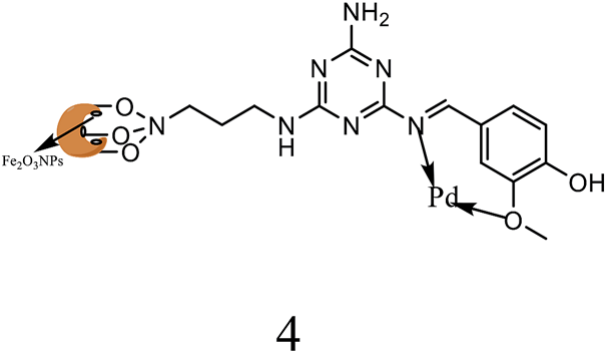	DMF, Et_3_N	40 min	120	97	45*h*
11	C_6_H_5_I + styrene	1	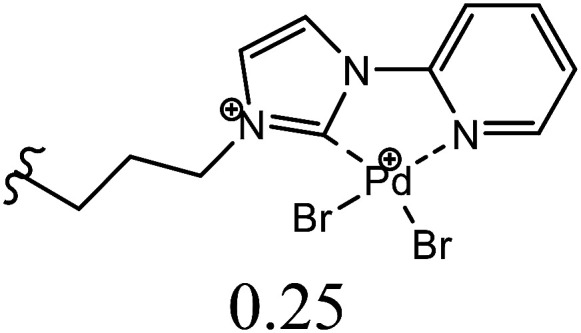	NMP, K_2_CO_3_	2.5	120	96	45*c*
12	C_6_H_5_I + styrene	400	Pd (OAc)_2_ : [H_2_L^1^][TfO]_2_ (1 : 1)	DMF, NaOAc	4	130	98	This work
13	C_6_H_5_Br + alkyl acrylate	1	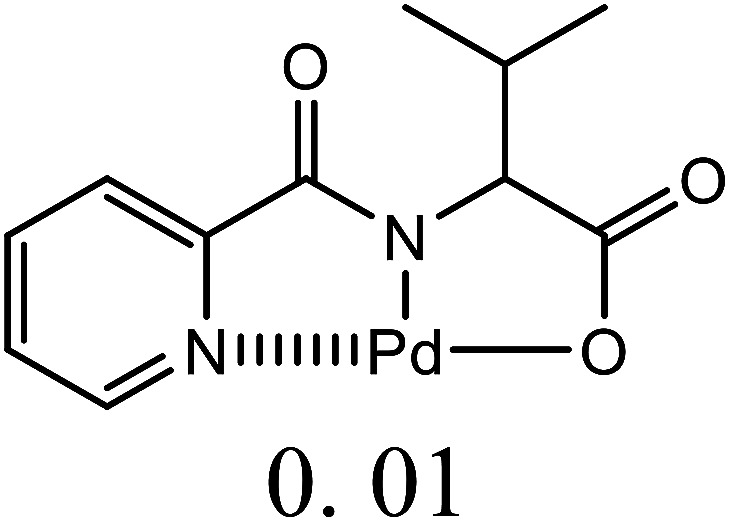	DMF, Na_2_CO_3_	20	145	92	45*f*
14	C_6_H_5_Br + alkyl acrylate	1	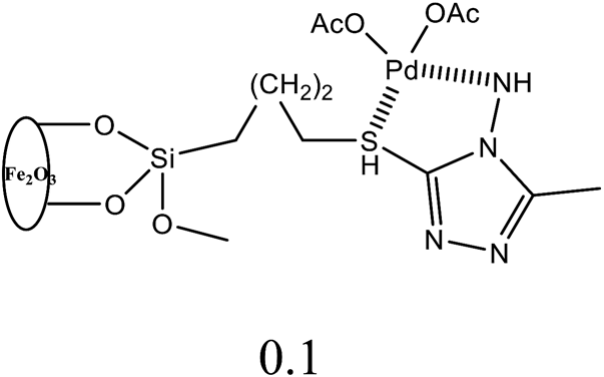	H_2_O, K_2_CO_3_	5	50	95	45*i*
15	C_6_H_5_Br + alkyl acrylate	400	Pd (OAc)_2_ : [H_2_L^1^][TfO]_2_ (1 : 1)	DMF, NaOAc	3.5	130	96	This work

a TBAI = tetrabutylammonium iodide, TEA = triethylamine, NMP = *N*-methyl-2-pyrrolidone.

We observed a probable limitation of our new catalytic system entry 12 as it was not found to be the best for the Heck coupling of iodobenzenes with styrene. The published literature ^45*c*,*h*^ (entry 10 and 11) proved it inferior to them with respect to catalyst loading but it still looks reasonable when compared to entries 7–9 (ref. 45*a*,*f*,*g*) of the above table.

The comparison of our result with literature with regard to the Heck coupling of bromobenzene with alkyl acrylate (entries 13–15) ^45*f*,*i*,*j*^ suggested that the catalyst system generated in this work (entry 19) proved to be an excellent attempt specially with respect to very less time utilization (3.5 hours). Likhar *et al.*,^45*f*^ got 92% of coupled product with 0.01 mol% but in 20 h and at 145 °C. In 2017, Abdol R. Hajipour^45*i*^ synthesized highly active catalyst and got 95% yield at only 50 °C but using more time than our catalytic system *i.e.* 5 hours. Wang *et al.*, ^45*j*^ used 0.005 mol% of catalyst but with only 4% yield. The other remarkable feature of our catalyst (entry 16) was the use of 400 mmol of halobenzene transformed into 96% of coupled product compared to literature (1 mmol) (Table 9).

## 4. Conclusion

In conclusion, five new ligands, [H_2_L^1^][OTf]_2_–[H_2_L^5^][I]_2_, were synthesized from two new precursors, [P^3^_Et_][I] and [P^2^_Me_][CF_3_SO_3_]. These ligands were successfully synthesized by the melt reaction between precursor and corresponding diamine. All the synthesized compounds were successfully characterized by various analytical techniques such as ^1^H and ^13^C NMR, FTIR, single crystal XRD and DFT studies. These ligands acted very efficiently as co-catalysts for palladium acetate in Heck–Mizoroki cross coupling reactions. These ligand systems are useful addition in the field of electron donor ligands such as NHC and phosphines that can stabilize palladium in different oxidation states during C–C coupling reactions.

## Conflicts of interest

There are no conflicts of interest among authors to declare.

## Supplementary Material

RA-009-C9RA07912B-s001

RA-009-C9RA07912B-s002
