# Supplementary material for: Synthesis of new Pro-PYE ligands as co-catalysts toward Pd-catalyzed Heck–Mizoroki cross coupling reactions
Source: RSC Adv. 2019 Nov 21;9(65):37986–8000. doi: 10.1039/c9ra07912b (PMC9075740; doi:10.1039/c9ra07912b)
Supplement: RA-009-C9RA07912B-s001 [file RA-009-C9RA07912B-s001.pdf]

Electronic Supplementary Material (ESI) for RSC Advances.
This journal is © The Royal Society of Chemistry 2019

Synthesis of New Pro-PYE Ligands as Co-Catalysts toward Pd-Catalyzed Heck-Mizoroki Cross Coupling Reactions

Naima Munir[1], Muhammad Naveed Zafar[1*], Sara Masood[1], Faroha Liaqat[1], Muhammad Tahir[2], SammerYousuf[3], Saima Kalsoom[4], Ehsan Ullah Mughal[5], Sajjad Sumrra[5], Aneela Maalik[6*]

[1]*Department of Chemistry, Quaid-i-Azam University, Islamabad-45320, Pakistan.*
[2]*Department of Physics, University of Sargodha, Sargodha-40100, Pakistan*
[3]*H. E. J. Research Institute of Chemistry, International Centre for Chemical and Biological Sciences, University of Karachi-75270, Pakistan.*
[4]*Centre for Interdisciplinary Research in Basic Sciences (CIRBS), International Islamic University, Islamabad, Pakistan.*
[5]*Department of Chemistry, University of Gujrat, Gujrat-50700, Pakistan.*
[6]*Department of Chemistry, COMSATS University Islamabad, Islamabad Campus, Park Road, Islamabad-45550, Pakistan.*

*Correspondence: Aneela Maalik, aneela.maalik@comsats.edu.pk , Tel.: +923335490834; Muhammad Naveed Zafar, mnzafar@qau.edu.pk , Tel.: +923314503061

DR.NAVEED ZAFAR/NAIMA/NA-03_1HNMR_DMSO

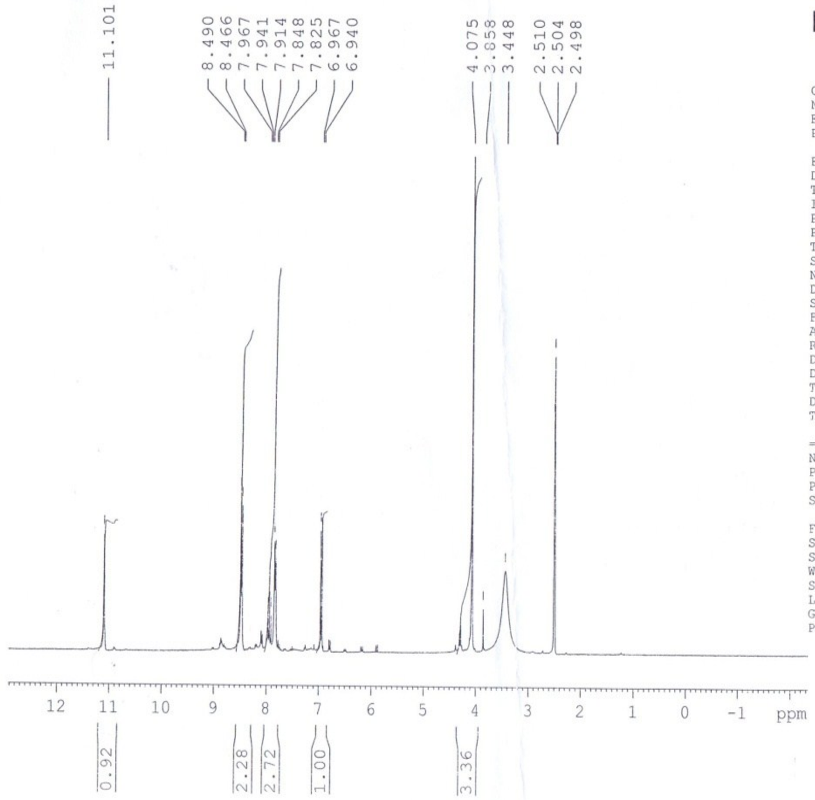

Current Data Parameters
NAME NA-03_1HNMR_DMSO
EXPNO 1
PROCNO 1

F2 - Acquisition Parameters
Date_ 20170804
Time 10.41
INSTRUM spect
PROBHD 5 mm BBO BB-1H
PULPROG zg30
TD 65536
SOLVENT DMSO
NS 8
DS 0
SWH 6172.839 Hz
FIDRES 0.094190 Hz
AQ 5.3084660 sec
RG 406.4
DW 81.000 usec
DE 6.00 usec
TE 296.4 K
D1 1.00000000 sec
TD0 1

======== CHANNEL f1 ========
NUC1 1H
P1 9.00 usec
PL1 2.00 dB
SFO1 300.1318534 MHz

F2 - Processing parameters
SI 32768
SF 300.1300000 MHz
WDW EM
SSB 0
LB 0.30 Hz
GB 0
PC 1.00

DR.NAVEED ZAFAR/NAIMA/NA-03P_13CNMR_DMSO

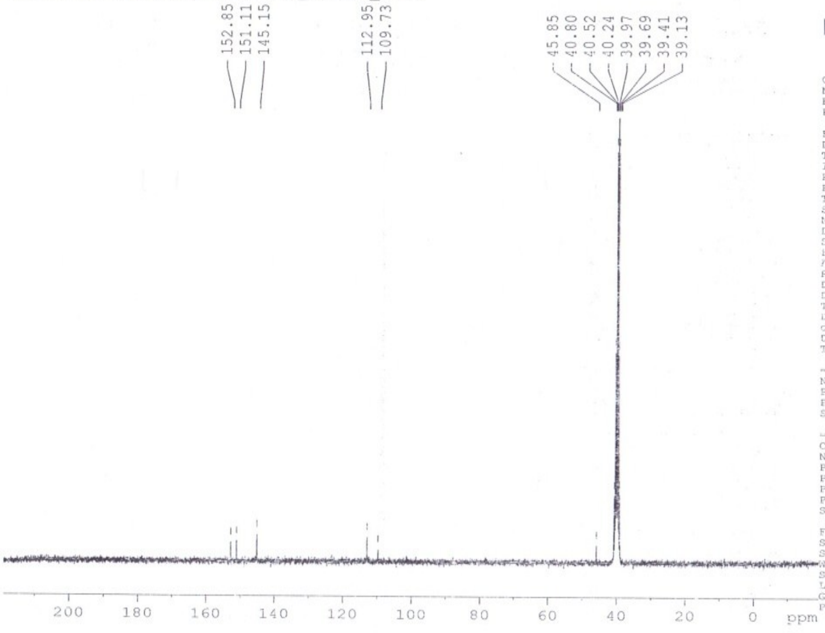

Current Data Parameters
NAME NA-03P_13CNMR_DMSO
EXPNO 1
PROCNO 1

F2 - Acquisition Parameters
Date_ 20171114
Time 13.03
INSTRUM spect
PROBHD 5 mm BBO BB-1H
PULPROG zgpg30
TD 35968
SOLVENT DMSO
NS 512
DS 0
SWH 17985.611 Hz
FIDRES 0.500045 Hz
AQ 0.9999604 sec
RG 3649.1
DW 27.800 usec
DE 6.00 usec
TE 294.4 K
D1 2.00000000 sec
d11 0.03000000 sec
DELTA 1.89999998 sec
TD0 1

======== CHANNEL f1 ========
NUC1 13C
P1 6.00 usec
PL1 -5.00 dB
SFO1 75.4752953 MHz

======== CHANNEL f2 ========
CPDPRG2 waltz16
NUC2 1H
PCPD2 80.00 usec
PL2 2.00 dB
PL12 20.98 dB
PL13 20.00 dB
SFO2 300.1312005 MHz

F2 - Processing parameters
SI 32768
SF 75.4677490 MHz
WDW EM
SSB 0
LB 1.00 Hz
GB 0
PC 1.40

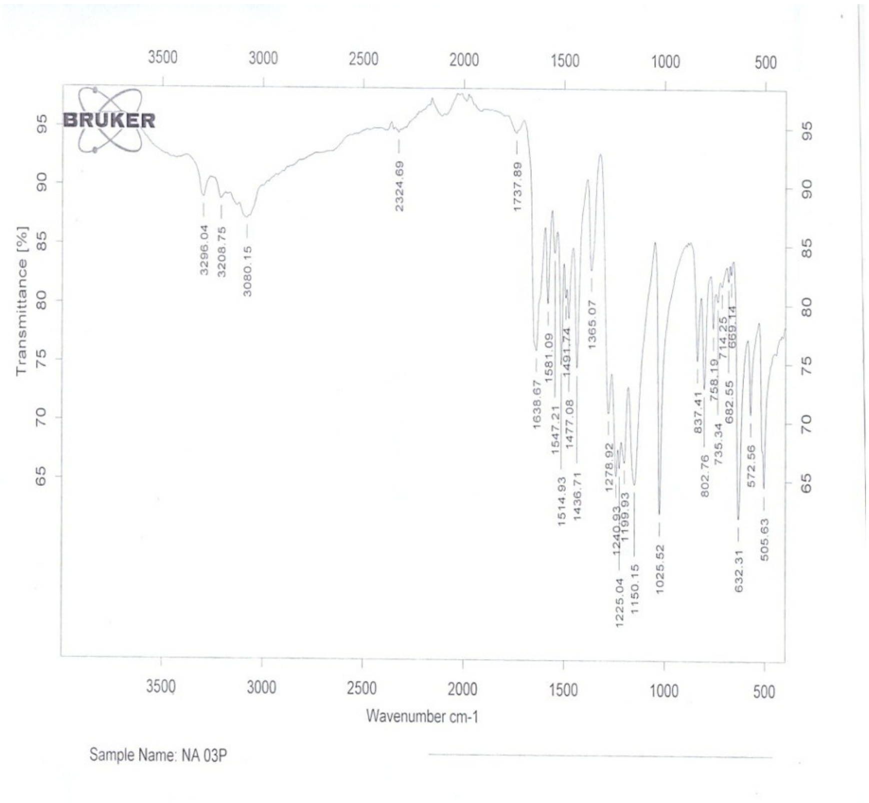

Figure S1: ^{1}H, ^{13}CNMR, IR spectra of $[H_2L^1][OTf]_2$

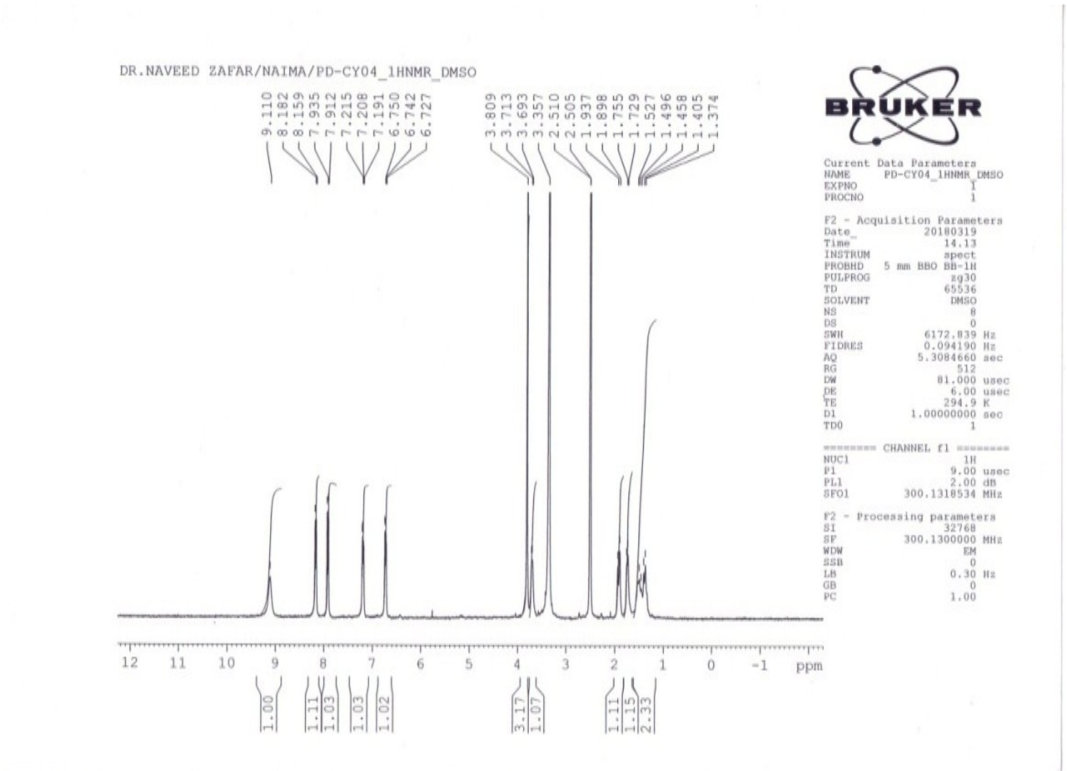

DR.NAVEED ZAFAR/NAIMA/PD-CY04_13CNMR_DMSO

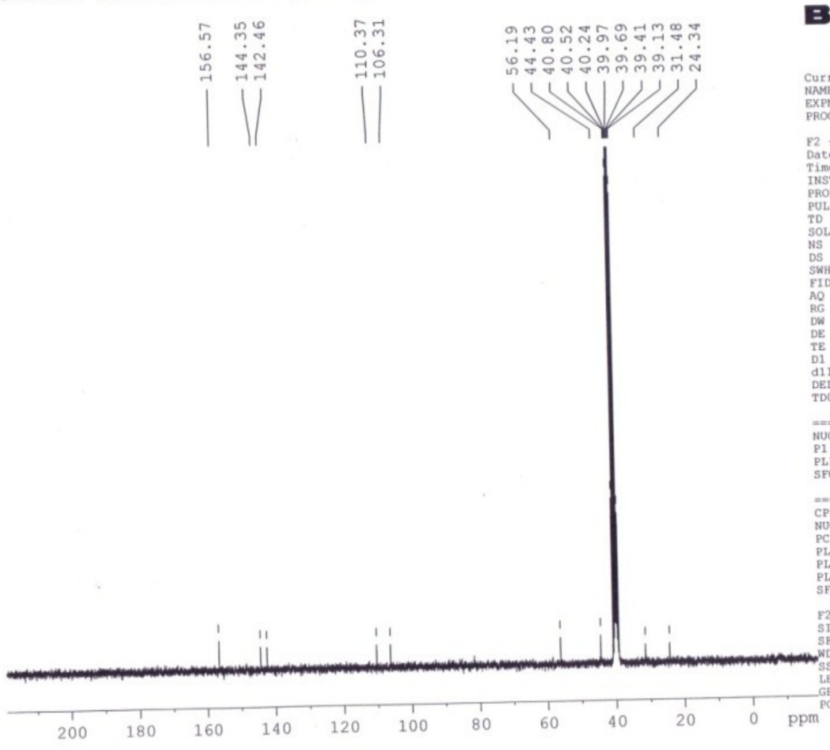

Current Data Parameters

NAME	PD-CY04_13CNMR_DMSO
EXPNO	1
PROCNO	1

F2 - Acquisition Parameters

Date_	20180319
Time	15.07
INSTRUM	spect
PROBHD	5 mm BBO BB-1H
PULPROG	zgpg30
TD	35968
SOLVENT	DMSO
NS	1024
DS	0
SWH	17985.611 Hz
FIDRES	0.500045 Hz
AQ	0.9999604 sec
RG	26008
DW	27.800 usec
DE	6.00 usec
TE	295.5 K
D1	2.00000000 sec
d11	0.03000000 sec
DELTA	1.89999998 sec
TD0	1

======== CHANNEL f1 ========

NUC1	13C
P1	6.00 usec
PL1	-5.00 dB
SFO1	75.4752953 MHz

======== CHANNEL f2 ========

CPDPRG2	waltz16
NUC2	1H
PCPD2	80.00 usec
PL2	2.00 dB
PL12	20.98 dB
PL13	20.00 dB
SFO2	300.1312005 MHz

F2 - Processing parameters

SI	32768
SF	75.4677490 MHz
WDW	EM
SSB	0
LB	1.00 Hz
GB	0
PC	1.40

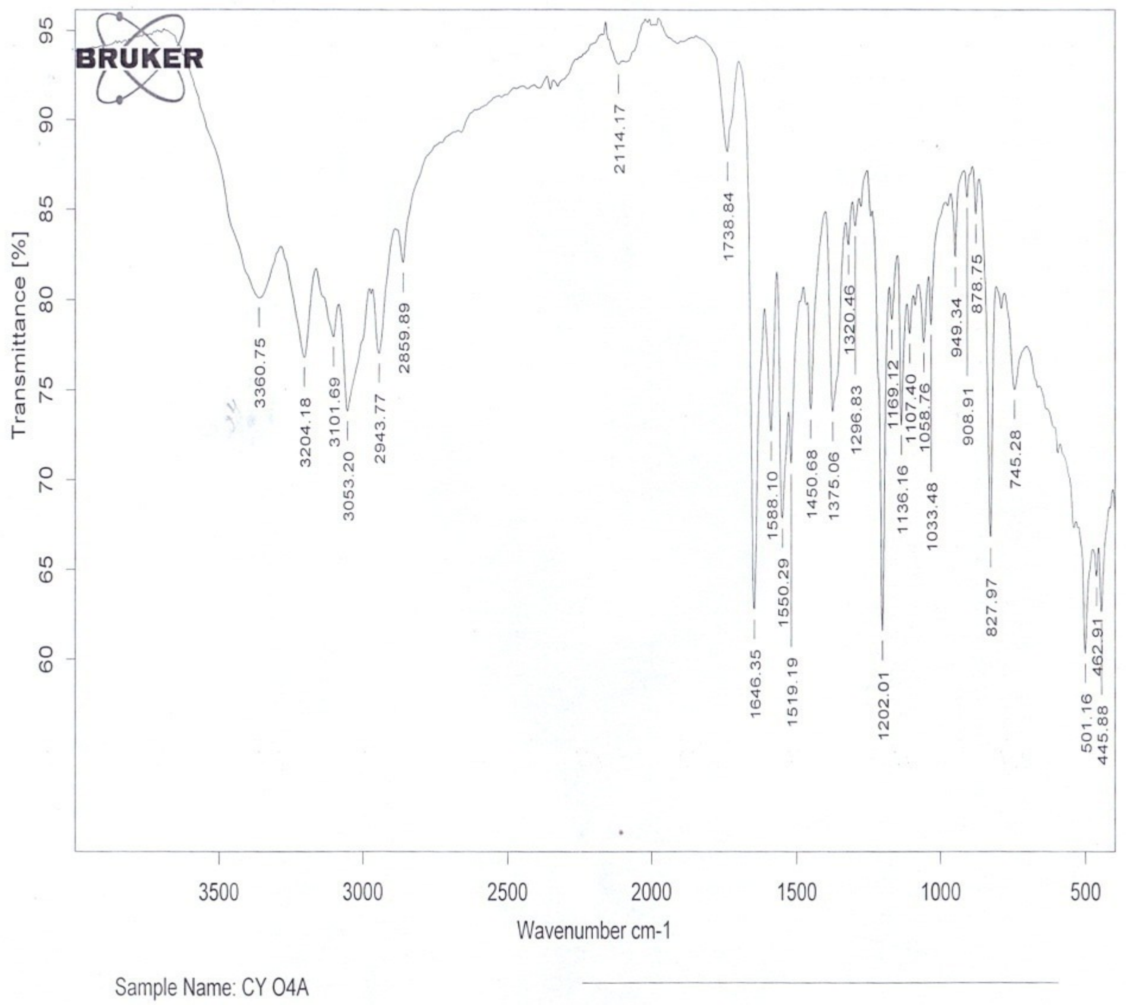

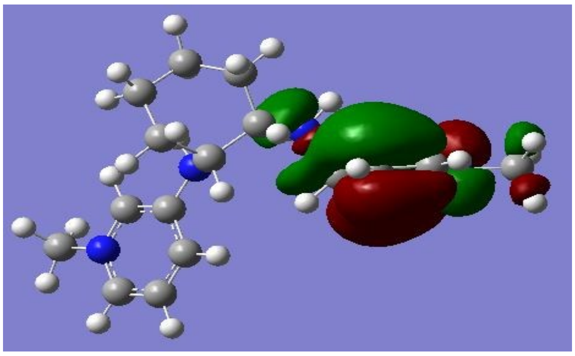

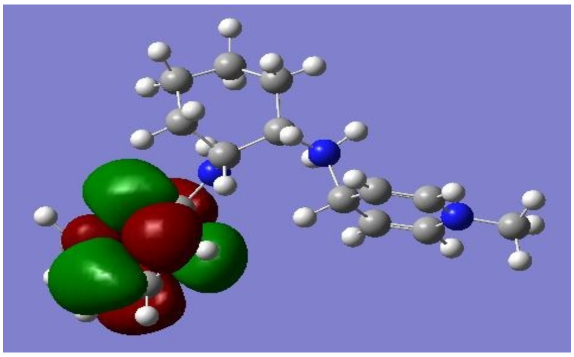

HOMO (Gaussian) **LUMO (Gaussian)**

Figure S2: ^{1}H, ^{13}CNMR, IR spectra and HOMO-LUMO of $[H_2L^2][OTf]_2$

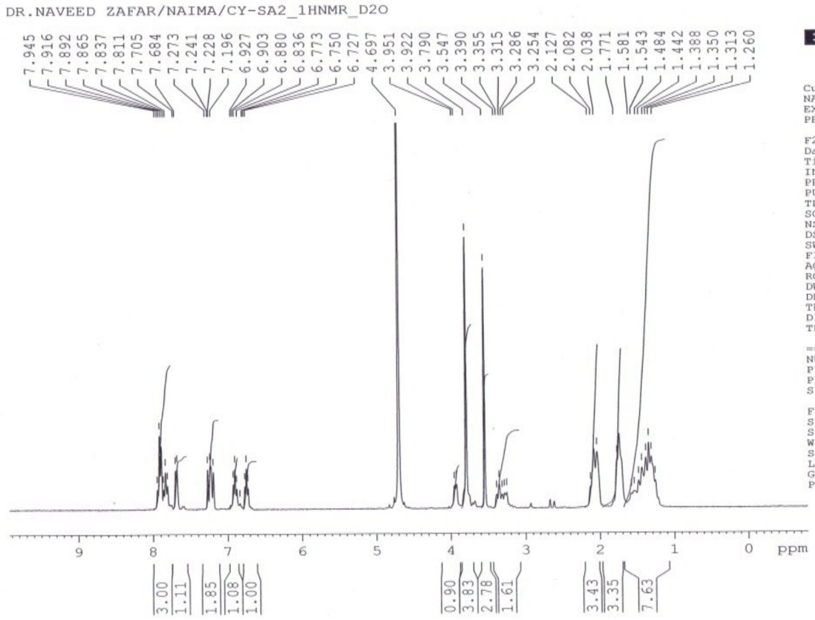

BRUKER

Current Data Parameters
NAME CY-SA2_1HNMR_D2O
EXPNO 1
PROCNO 1

F2 - Acquisition Parameters
Date_ 20180118
Time 14.33
INSTRUM spect
PROBHD 5 mm BBO BB-1H
PULPROG zg30
TD 65536
SOLVENT D2O
NS 8
DS 0
SWH 6172.839 Hz
FIDRES 0.094190 Hz
AQ 5.3084660 sec
RG 362
DW 81.000 usec
DE 6.00 usec
TE 293.5 K
D1 1.00000000 sec
TD0 1

======= CHANNEL f1 =======
NUC1 1H
P1 9.00 usec
PL1 2.00 dB
SFO1 300.1318534 MHz

F2 - Processing parameters
SI 32768
SF 300.1300000 MHz
WDW EM
SSB 0
LB 0.30 Hz
GB 0
PC 1.00

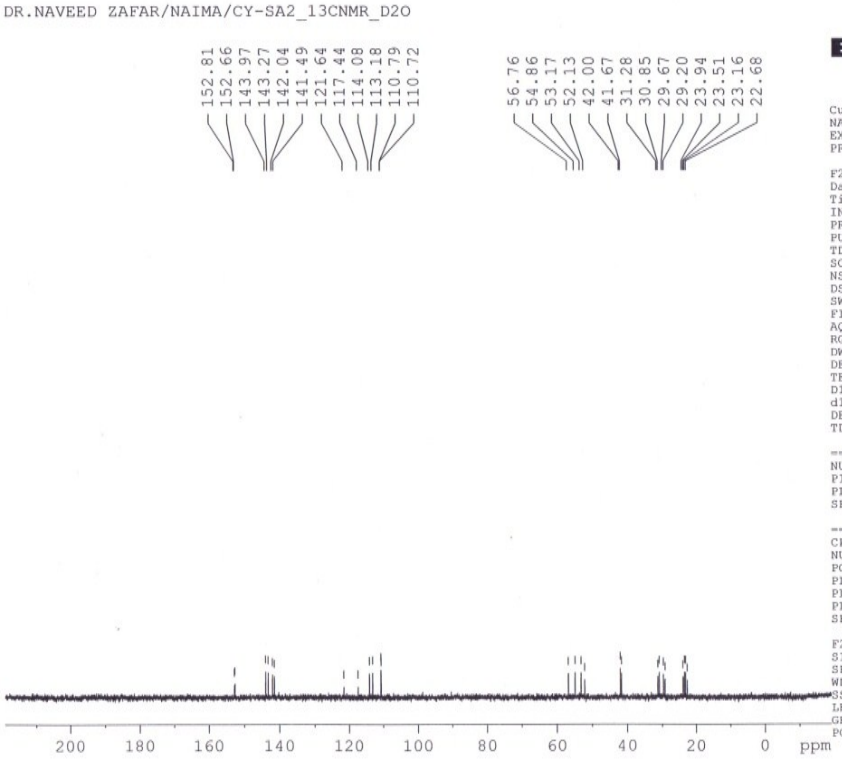

BRUKER

Current Data Parameters
NAME CY-SA2_13CNMR_D2O
EXPNO 1
PROCNO 1

F2 - Acquisition Parameters
Date_ 20180118
Time 15.26
INSTRUM spect
PROBHD 5 mm BBO BB-1H
PULPROG zgpg30
TD 35968
SOLVENT D2O
NS 1024
DS 0
SWH 17985.611 Hz
FIDRES 0.500045 Hz
AQ 0.9999604 sec
RG 32768
DW 27.800 usec
DE 6.00 usec
TE 294.0 K
D1 2.00000000 sec
d11 0.03000000 sec
DELTA 1.89999998 sec
TD0 1

======= CHANNEL f1 =======
NUC1 13C
P1 6.00 usec
PL1 -5.00 dB
SFO1 75.4752953 MHz

======= CHANNEL f2 =======
CPDPRG2 waltz16
NUC2 1H
PCPD2 80.00 usec
PL2 2.00 dB
PL12 20.98 dB
PL13 20.00 dB
SFO2 300.1312005 MHz

F2 - Processing parameters
SI 32768
SF 75.4677490 MHz
WDW EM
SSB 0
LB 1.00 Hz
GB 0
PC 1.40

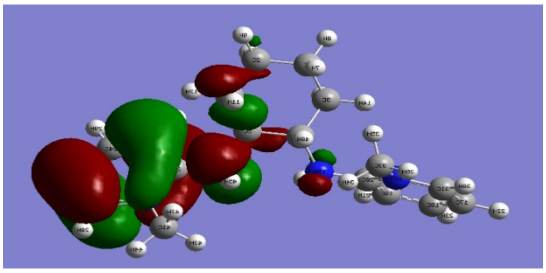

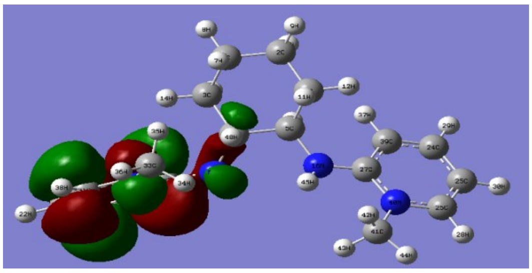

HOMO (Gaussian) **LUMO (Gaussian)**

Bond angles

Concerned Atoms	Experimental	Calculated	Concerned Atoms	Experimental	Calculated
C1-N1-C2	123.0	124.9	O1-S1-O3	114.8	113.2
N1-C2-C3	123.6	122.9	O2-S1-O1	114.3	113.8
C2-C3-C4	120.6	121.4	O3-S1-O2	115.3	105.1
C3-C4-C5	119.8	119.5	O1-S1-C8	103.7	106.2
C4-C5-C6	119.0	118.7	O3-S1-C8	103.8	106.5
C5-C6-N2	121.7	122.0	O2-S1-C8	102.7	106.4
C6-N2-C7	119.4	117.2	F3-C8-F1	107.1	110.1
C6-N2-C2	120.8	121.5	F2-C8-F3	106.0	111.3
N2-C2-C3	118.0	117.0	F1-C8-F2	105.1	109.5
C7-N2-C2	119.7	121.2	F2-C8-S1	112.0	106.0
N2-C2-N1	118.4	120.1	F3-C8-S1	111.8	108.5

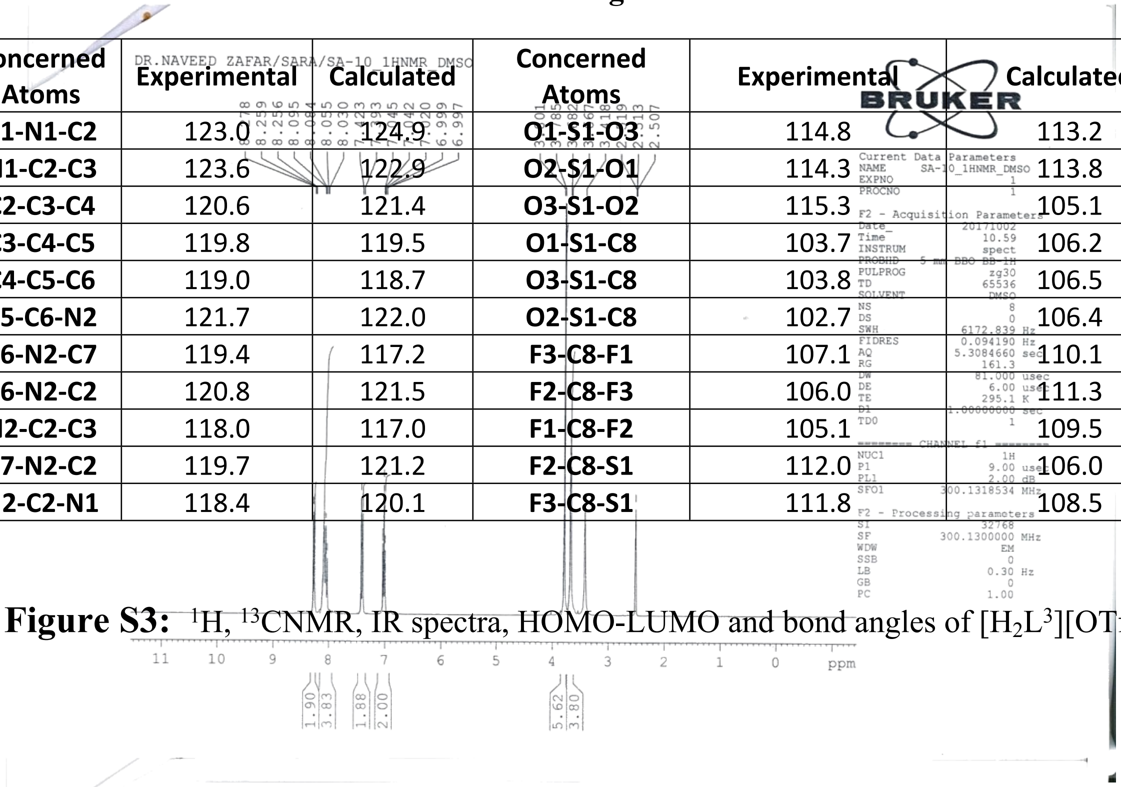

Figure S3: ^{1}H, ^{13}CNMR, IR spectra, HOMO-LUMO and bond angles of $[H_2L^3][OTf]_2$

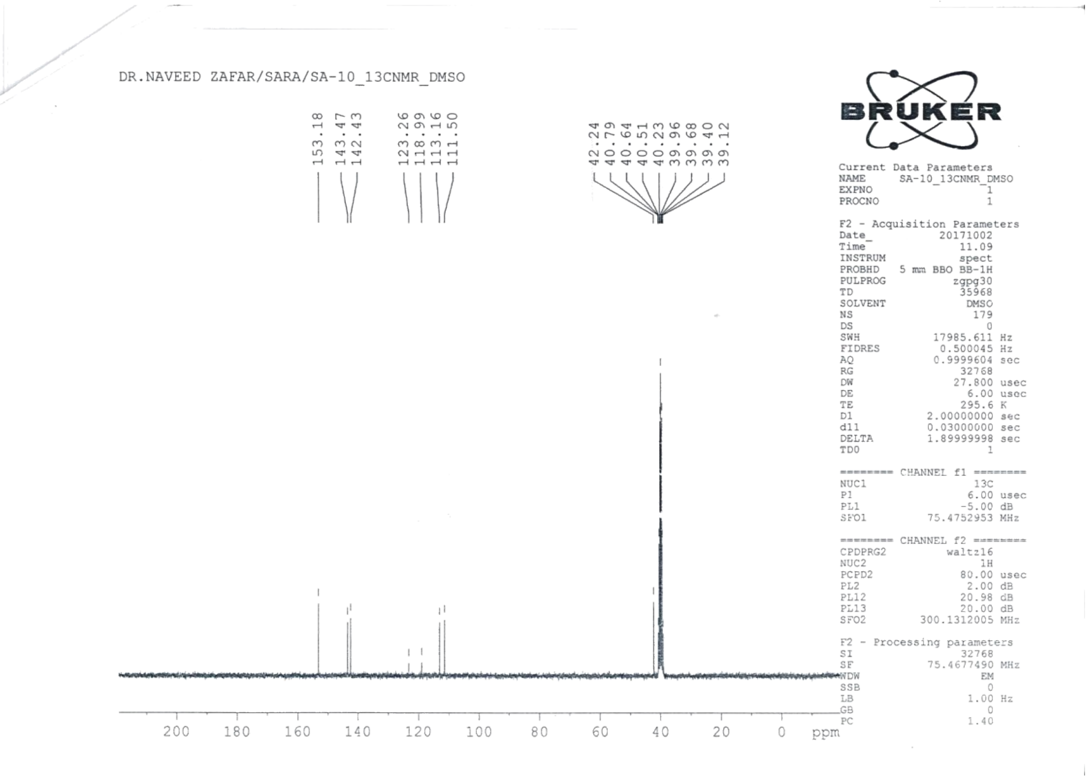

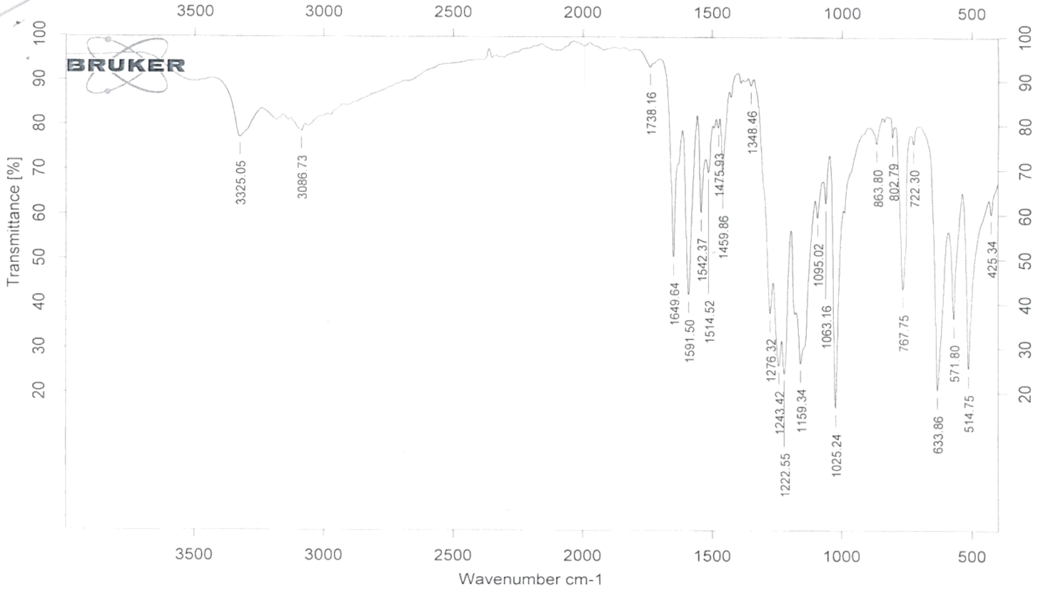

Sample Name: SRM 2B

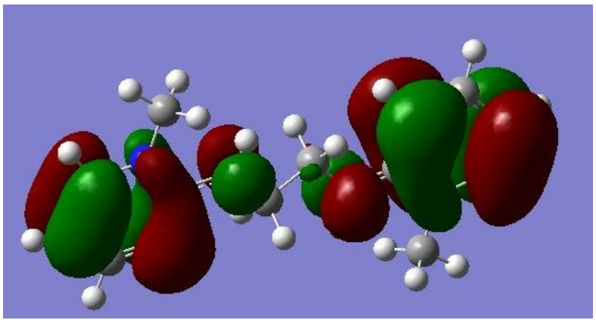

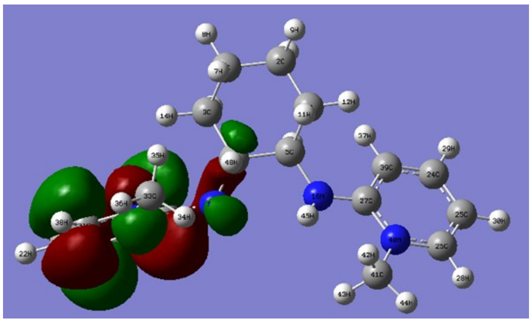

HOMO (Gaussian) **LUMO (Gaussian)**

Bond angles

Atom no's	Experimental	Calculated	Atom no's	Experimental	Calculated
C1-N1	1.46	1.49	O1-S1	1.43	1.47
N1-C2	1.34	1.39	O2-S1	1.43	1.39
C2-C3	1.40	1.41	S1-C8	1.82	1.82
C3-C4	1.36	1.39	F3-C8	1.31	1.37
C4-C5	1.39	1.38	F1-C8	1.33	1.36
C5-C6	1.34	1.38	F2-C8	1.32	1.36
C6-N2	1.36	1.35	C1-C1	1.51	2.19
N2-C7	1.46	1.48	N2-C2	1.36	1.38
N2-C2	1.36	1.38	O3-SI	1.42	1.47
O3-SI	1.42	1.47	O1-S1	1.43	1.47

Figure S4: ^{1}H, ^{13}CNMR, IR spectra, HOMO-LUMO & bond angles of $[H_2L^4][OTf]_2$

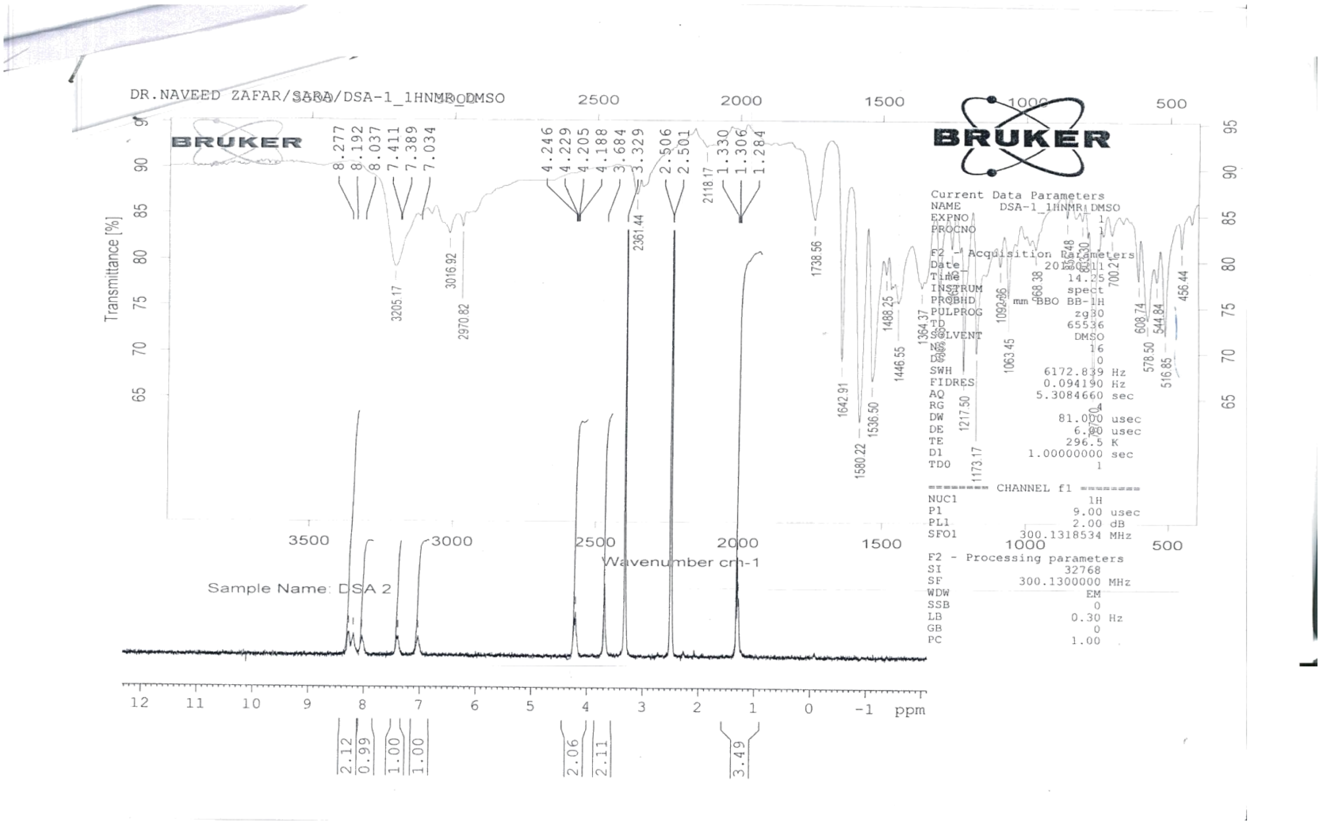

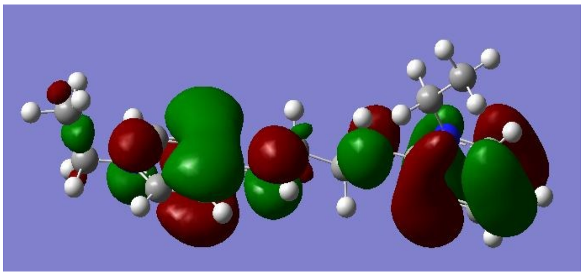

HOMO (Gaussian)

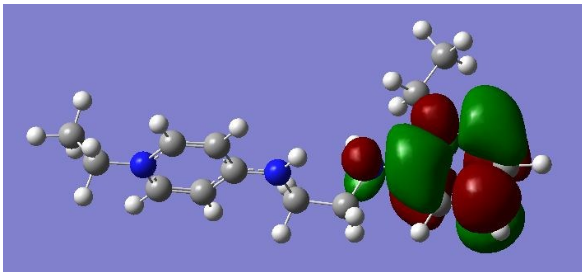

LUMO (Gaussian)

Bond angles

Concerned Atoms	Experimental	Calculated
**C1-N1-C2**	123.7	122.9
**N1-C2-C3**	123.5	121.7
**C2-C3-C4**	121.2	121.4
**C3-C4-C5**	119.6	119.3
**C4-C5-C6**	119.0	118.8
**C5-C6-N2**	122.7	122.1
**C6-N2-C2**	120.0	121.2
**N2-C2-C3**	117.4	117.3
**N2-C2-N1**	119.1	121.0
**C6-N2-C7**	120.1	116.9
**N2-C7-C8**	110.0	109.5

Figure S5: ^{1}H, ^{13}CNMR, IR spectra, HOMO-LUMO and bond angles of $[H_2L^5][I]_2$

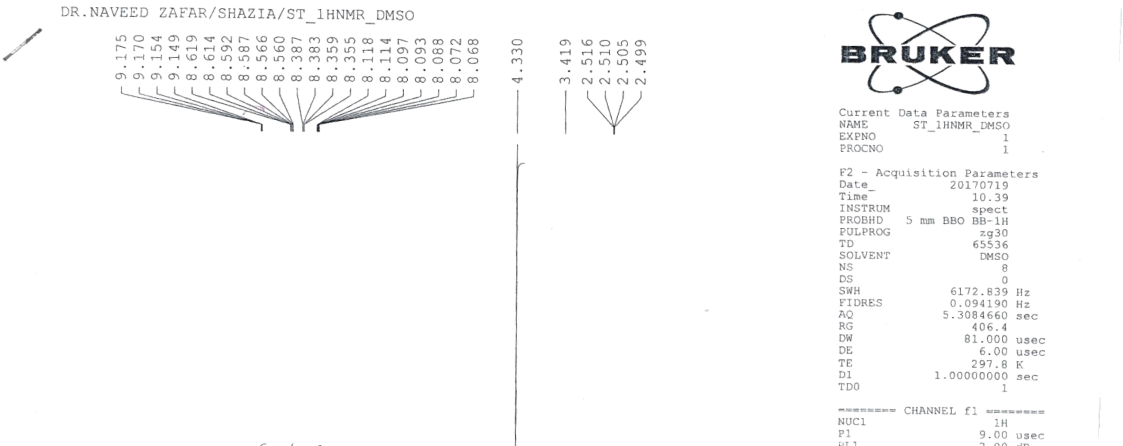

DR.NAVEED ZAFAR/SHAZIA/ST_13CNMR_DMSO

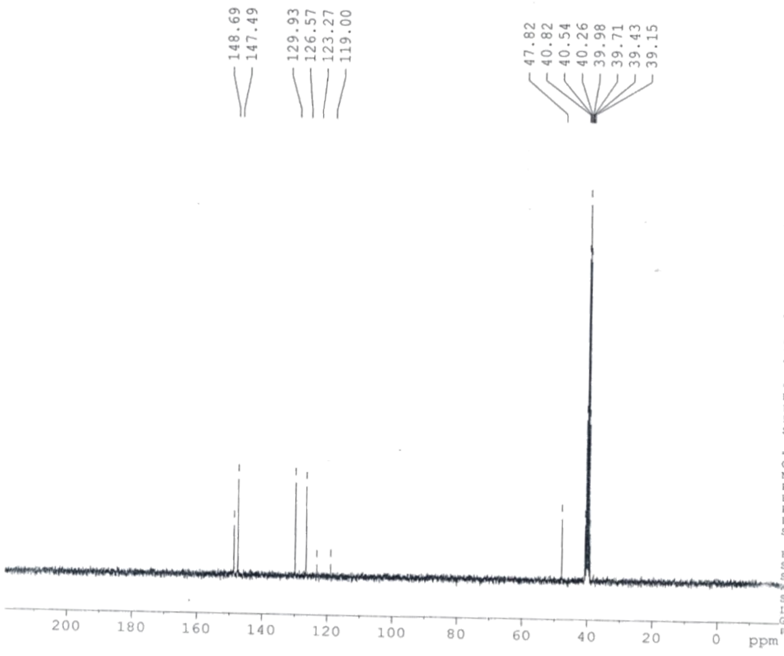

**Current Data Parameters**		
NAME	ST_13CNMR_DMSO	
EXPNO	1	
PROCNO	1	
**F2 - Acquisition Parameters**		
Date_	20170719	
Time	10.50	
INSTRUM	spect	
PROBHD	5 mm BBO BB-1H	
PULPROG	zgpg30	
TD	35968	
SOLVENT	DMSO	
NS	176	
DS	0	
SWH	17985.611	Hz
FIDRES	0.500045	Hz
AQ	0.9999604	sec
RG	13004	
DW	27.800	usec
DE	6.00	usec
TE	300.0	K
D1	2.00000000	sec
d11	0.03000000	sec
DELTA	1.89999998	sec
TD0	1	
**======== CHANNEL f1 ========**		
NUC1	13C	
P1	6.00	usec
PL1	-5.00	dB
SFO1	75.4752953	MHz
**======== CHANNEL f2 ========**		
CPDPRG2	waltz16	
NUC2	1H	
PCPD2	80.00	usec
PL2	2.00	dB
PL12	20.98	dB
PL13	20.00	dB
SFO2	300.1312005	MHz
**F2 - Processing parameters**		
SI	32768	
SF	75.4677490	MHz
WDW	EM	
SSB	0	
LB	1.00	Hz
GB	0	
PC	1.40	

Bond angles

Concerned Atoms	Experimental	Calculated	Concerned Atoms	Experimental	Calculated
**Cl_1-C_1-N_1**	117.5	117.4	**H6B-C6-H6C**	109.5	109.5
**C_6-N_1-C_5**	118.7	118.8	**H6B-C6-H6A**	109.5	109.5
**C_1-N_1-C_5**	119.3	120.3	**H6A-C6-H6C**	109.5	109.5
**C_1-N_1-C_6**	121.9	120.6	**H6C-C6-N1**	109.5	109.5
**C_4-C_5-N_1**	121.3	121.3	**H6B-C6-N1**	109.5	109.5
**N_1-C_1-C_2**	121.2	119.6	**H6A-C6-N1**	109.5	109.5
**C_5-C_4-C_3**	119.4	119.0	**F1-C7-S1**	112.0	111.9
**C_4-C_3-C_2**	119.7	119.5	**F3-C7-S1**	111.4	111.6
**C_3-C_2-C_1**	119.0	119.0	**O2-S1-O1**	114.0	113.9

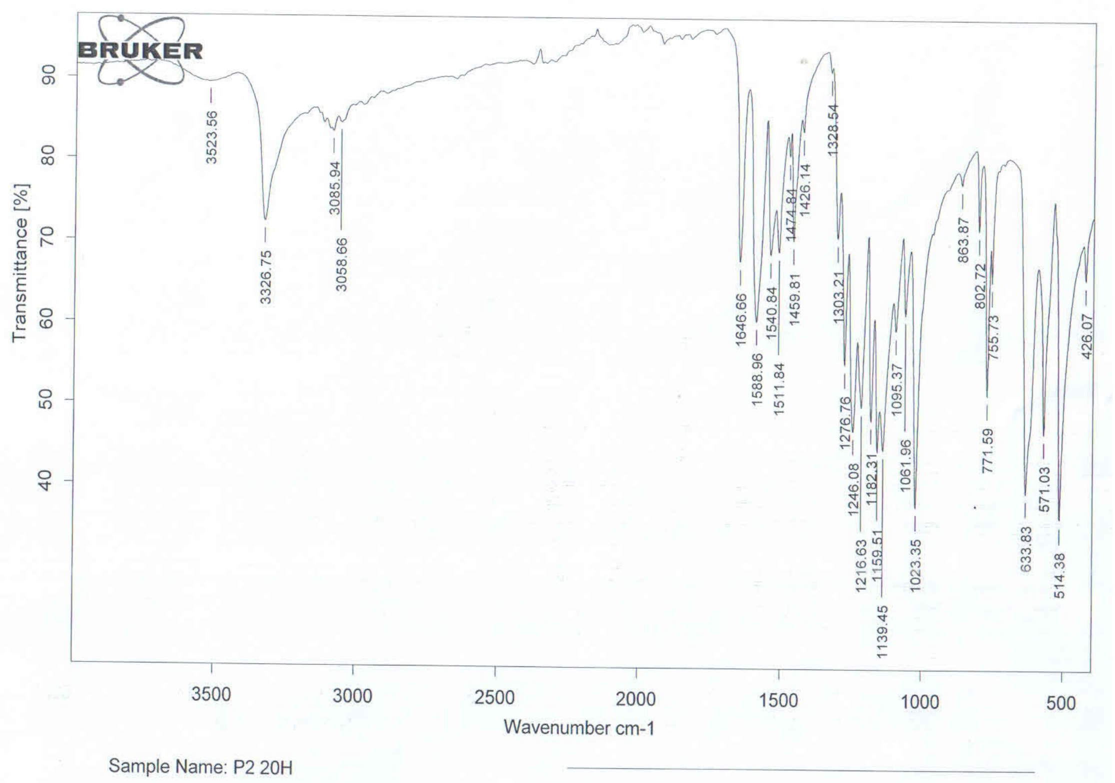

**Cl1-C1-C2**	121.3	118.1	**O3-S1-O1**	115.1	115.0
**C7-S1-O2**	103.3	106.4	**N1-C5-H5**	119.4	119.2
**C7-S1-O3**	103.4	106.5	**H5-C5-C4**	119.3	119.1
**C7-S1-O1**	103.1	106.4	**H2-C2-C3**	120.5	120.4
**F1-C7-F3**	108.4	112.5	**H2-C2-C1**	120.5	120.4
**F3-C7-F2**	109.6	113.4	**C2-C3-H3**	120.2	120.1
**F1-C7-F2**	106.5	105.7	**H3-C3-C4**	120.1	120.1
**F2-C7-S1**	112.6	107.6	**C5-C4-H4**	120.3	120.4
**O2-S1-O3**	115.6	114.5	**C3-C4-H4**	120.3	120.4

Bond lengths

**Concerned Atoms**	**Experimental**	**Calculated**	**Concerned Atoms**	**Experimental**	**Calculated**
**Cl1-C1**	1.69	1.70	**S1-O3**	1.42	1.39
**C1-C2**	1.37	1.39	**S1-O2**	1.43	1.40
**C2-C3**	1.37	1.39	**S1-O1**	1.43	1.40
**C3-C4**	1.37	1.37	**N1-C1**	1.34	1.33
**C4-C5**	1.36	1.36	**C5-H5**	0.93	0.95
**N1-C5**	1.35	1.35	**C2-H2**	0.93	0.95
**C6-N1**	1.48	1.47	**C3-H3**	0.93	0.95
**C7-S1**	1.80	1.76	**C4-H4**	0.93	0.95
**C7-F2**	1.32	1.30	**C6-H6B**	0.96	1.00
**C7-F3**	1.32	1.31	**C6-H6A**	0.96	1.00
**C7-F1**	1.32	1.33	**C6-H6C**	0.96	1.00

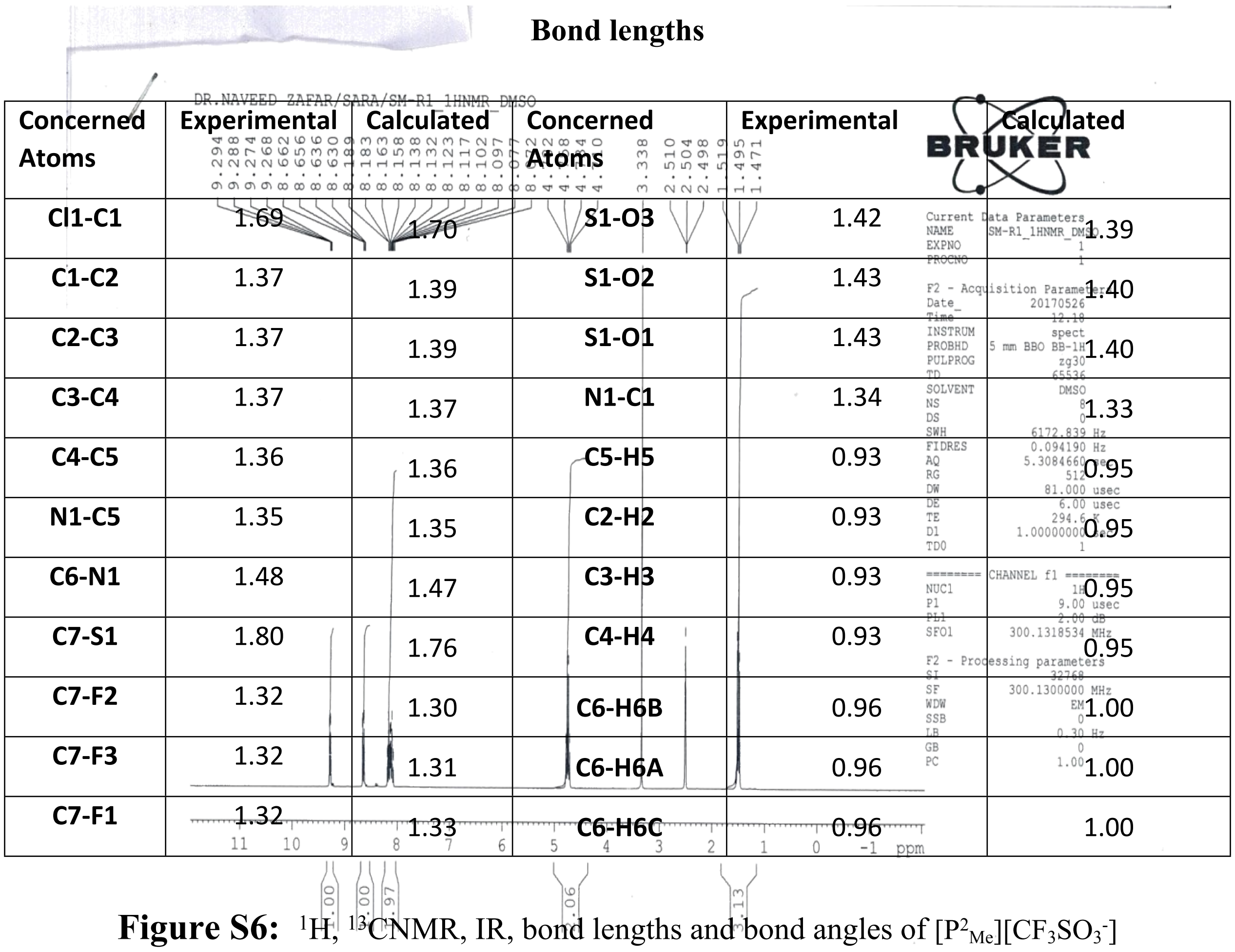

Figure S6: ^{1}H, ^{13}CNMR, IR, bond lengths and bond angles of $[P^{2}_{Me}][CF_3SO_3^-]$

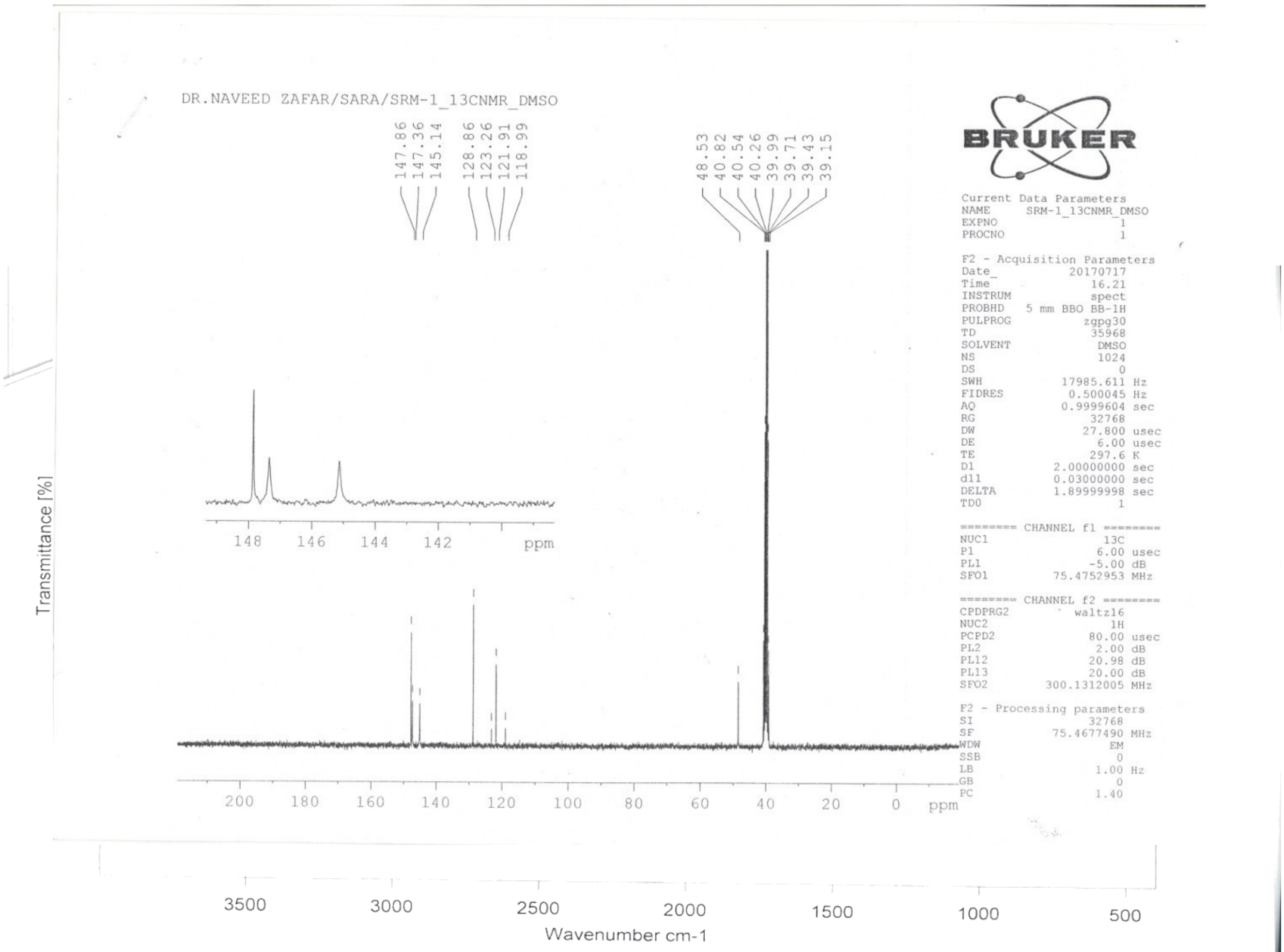

Sample Name: SRE 1

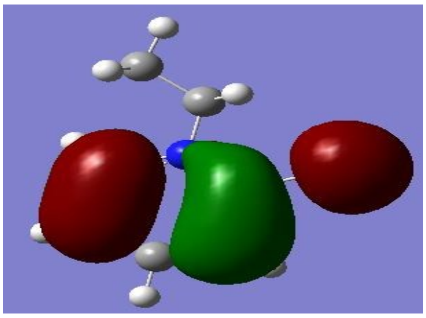

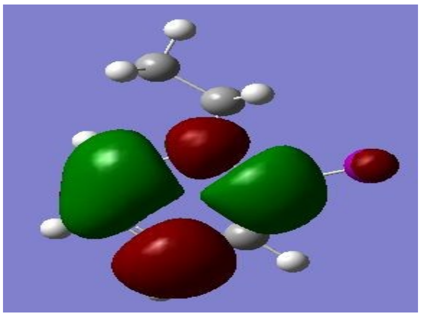

HOMO (Gaussian) **LUMO (Gaussian)**

Bond angles

Concerned Atoms	Experimental	Calculated	Concerned Atoms	Experimental	Calculated
**C5-C4-C3**	114.5	119.2	**C4-C5-H5**	114.2	114.1
**C4-C3-C2**	113.7	119.2	**N1-C6-H6A**	110.0	109.1
**C3-C2-C1**	130.6	120.3	**N1-C6-H6B**	109.9	108.7
**I1-C1-N1**	117.7	125.5	**N1-C6-C7**	109.1	108.3
**N1-C5-C4**	131.5	121.6	**H6A-C6-H6B**	108.2	107.8
**I1-C1-C2**	127.7	115.1	**H6A-C6-C7**	109.9	108.9
**N1-C6-C7**	109.1	109.8	**H6B-C6-C7**	109.8	108.9
**C1-N1-C6**	114.7	115.4	**C6-C7-H7A**	109.5	108.7
**C5-N1-C6**	130.1	131.0	**C6-C7-H7B**	109.5	108.7

**C1-I1-I2**	177.5	178.4	**C6-C7-H7C**	109.5	108.7
**C1-C2-H2**	114.6	114.5	**H7A-C7-H7B**	109.5	108.7
**H2-C2-C3**	114.8	113.9	**N1-C1-C2**	114.6	114.4
**C2-C3-H3**	123.1	122.4	**H7A-C7-H7C**	109.5	108.9
**H3-C3-C4**	123.2	122.5	**H7B-C7-H7C**	109.4	108.9

Bond lengths

Concerned Atoms	Experimental values	Computed values	Concerned Atoms	Experimental values	Computed values
**N1-C1**	1.09	1.10	**C1-I1**	2.02	2.12
**C1-C2**	1.31	1.36	**C2-H**	0.93	1.00
**C2-C3**	1.33	1.35	**C3-H**	0.93	1.00
**C3-C4**	1.12	1.23	**C4-H**	0.93	1.00
**C4-C5**	1.31	1.35	**C5-H**	0.93	1.00
**N1-C6**	1.43	1.43	**C6-H**	0.97	1.02
**C6-C7**	1.24	1.25	**C6-H**	0.97	1.01
**N1- C5**	1.29	1.34	**C7-H**	0.96	1.00
**I1-I2**	3.24	3.36	**C7-H**	0.96	1.00

Figure S7: ^{1}H, ^{13}CNMR, IR spectra, HOMO LUMO, bond lengths and bond angles of $[P^3_{Et}][I^-]$

Table 1: Crystal structure Data of S3

Table 1: Crystal data and structure refinement for a.

Identification code	8A
Empirical formula	C10 H13 F3 N2 O3 S
Formula weight	298.28
Temperature	100(2) K
Wavelength	1.54178 Å

Crystal system	Orthorhombic	
Space group	Pbcn	
Unit cell dimensions	a = 15.5634(3) Å	α= 90°.
	b = 12.4382(2) Å	β= 90°.
	c = 12.8282(3) Å	γ = 90°.
Volume	2483.29(9) $Å^3$	
Z	8	
Density (calculated)	1.596 Mg/m^3	
Absorption coefficient	2.780 mm^{-1}	
F(000)	1232	
Crystal size	0.170 x 0.050 x 0.040 mm^3	
Theta range for data collection	4.551 to 68.305°.	
Index ranges	-18<=h<=18, -14<=k<=14, -15<=l<=15	
Reflections collected	36806	
Independent reflections	2282 [R(int) = 0.0771]	
Completeness to theta = 67.679°	100.0 %	
Refinement method	Full-matrix least-squares on F^2	
Data / restraints / parameters	2282 / 0 / 177	
Goodness-of-fit on F^2	1.049	
Final R indices [I>2sigma(I)]	R1 = 0.0580, wR2 = 0.1377	
R indices (all data)	R1 = 0.0684, wR2 = 0.1446	
Extinction coefficient	n/a	
Largest diff. peak and hole	1.159 and -0.746 e.$Å^{-3}$	

Atomic coordinates (x 10^4) and equivalent isotropic displacement parameters ($Å^2$x 10^3) for a. U(eq) is defined as one third of the trace of the orthogonalized U^{ij} tensor.

	x	y	z	U(eq)
S(1)	2443(1)	6040(1)	5767(1)	17(1)
F(1)	894(2)	5291(2)	5554(4)	104(2)
F(2)	1788(1)	4081(2)	5875(2)	32(1)
F(3)	1746(3)	4798(3)	4369(2)	99(2)
O(1)	2345(2)	6101(2)	6870(2)	50(1)
O(2)	3253(2)	5576(2)	5474(3)	46(1)
O(3)	2196(2)	6945(2)	5156(2)	32(1)
N(1)	4128(2)	4665(2)	2998(2)	14(1)
N(2)	3768(2)	6459(2)	3240(2)	16(1)
C(1)	3932(2)	7474(3)	3596(3)	23(1)
C(2)	4672(2)	7707(3)	4100(3)	27(1)
C(3)	5262(2)	6879(3)	4275(2)	21(1)
C(4)	5089(2)	5857(3)	3949(2)	16(1)
C(5)	4325(2)	5635(2)	3398(2)	13(1)
C(6)	2971(2)	6281(3)	2649(3)	22(1)
C(7)	4699(2)	3727(2)	2979(2)	13(1)
C(8)	4142(2)	2714(2)	2962(3)	17(1)
C(9)	4689(2)	1693(3)	2960(3)	21(1)
C(10)	1688(2)	4992(3)	5367(3)	28(1)

Bond lengths [Å] and angles [A°] for a.

S(1)-O(3)	1.425(3)

S(1)-O(1)	1.426(3)
S(1)-O(2)	1.437(3)
S(1)-C(10)	1.828(4)
F(1)-C(10)	1.313(5)
F(2)-C(10)	1.315(4)
F(3)-C(10)	1.306(5)
N(1)-C(5)	1.346(4)
N(1)-C(7)	1.468(4)
N(1)-H(5)	0.84(4)
N(2)-C(5)	1.358(4)
N(2)-C(1)	1.367(4)
N(2)-C(6)	1.470(4)
C(1)-C(2)	1.352(5)
C(1)-H(1)	0.9500
C(2)-C(3)	1.398(5)
C(2)-H(2)	0.9500
C(3)-C(4)	1.366(5)
C(3)-H(3)	0.9500
C(4)-C(5)	1.412(4)
C(4)-H(4)	0.9500
C(6)-H(6A)	0.9800
C(6)-H(6B)	0.9800
C(6)-H(6C)	0.9800
C(7)-C(8)	1.529(4)
C(7)-C(7)#1	1.546(6)

C(7)-H(7)	1.0000
C(8)-C(9)	1.528(4)
C(8)-H(8A)	0.9900
C(8)-H(8B)	0.9900
C(9)-C(9)#1	1.526(7)
C(9)-H(9A)	0.9900
C(9)-H(9B)	0.9900

O(3)-S(1)-O(1)	118.35(19)
O(3)-S(1)-O(2)	114.25(18)
O(1)-S(1)-O(2)	112.0(2)
O(3)-S(1)-C(10)	103.68(16)
O(1)-S(1)-C(10)	104.37(18)
O(2)-S(1)-C(10)	101.78(18)
C(5)-N(1)-C(7)	125.5(3)
C(5)-N(1)-H(5)	121(3)
C(7)-N(1)-H(5)	113(3)
C(5)-N(2)-C(1)	121.9(3)
C(5)-N(2)-C(6)	120.1(3)
C(1)-N(2)-C(6)	118.0(3)
C(2)-C(1)-N(2)	121.2(3)
C(2)-C(1)-H(1)	119.4
N(2)-C(1)-H(1)	119.4
C(1)-C(2)-C(3)	118.6(3)
C(1)-C(2)-H(2)	120.7

C(3)-C(2)-H(2)	120.7
C(4)-C(3)-C(2)	120.5(3)
C(4)-C(3)-H(3)	119.8
C(2)-C(3)-H(3)	119.8
C(3)-C(4)-C(5)	120.1(3)
C(3)-C(4)-H(4)	119.9
C(5)-C(4)-H(4)	119.9
N(1)-C(5)-N(2)	118.3(3)
N(1)-C(5)-C(4)	123.9(3)
N(2)-C(5)-C(4)	117.7(3)
N(2)-C(6)-H(6A)	109.5
N(2)-C(6)-H(6B)	109.5
H(6A)-C(6)-H(6B)	109.5
N(2)-C(6)-H(6C)	109.5
H(6A)-C(6)-H(6C)	109.5
H(6B)-C(6)-H(6C)	109.5
N(1)-C(7)-C(8)	108.2(2)
N(1)-C(7)-C(7)#1	112.4(2)
C(8)-C(7)-C(7)#1	109.4(2)
N(1)-C(7)-H(7)	109.0
C(8)-C(7)-H(7)	109.0
C(7)#1-C(7)-H(7)	109.0
C(9)-C(8)-C(7)	111.7(3)
C(9)-C(8)-H(8A)	109.3
C(7)-C(8)-H(8A)	109.3

C(9)-C(8)-H(8B)	109.3
C(7)-C(8)-H(8B)	109.3
H(8A)-C(8)-H(8B)	107.9
C(9)#1-C(9)-C(8)	110.8(2)
C(9)#1-C(9)-H(9A)	109.5
C(8)-C(9)-H(9A)	109.5
C(9)#1-C(9)-H(9B)	109.5
C(8)-C(9)-H(9B)	109.5
H(9A)-C(9)-H(9B)	108.1
F(3)-C(10)-F(1)	107.2(4)
F(3)-C(10)-F(2)	108.6(3)
F(1)-C(10)-F(2)	105.4(3)
F(3)-C(10)-S(1)	111.3(3)
F(1)-C(10)-S(1)	110.6(3)
F(2)-C(10)-S(1)	113.5(2)

Symmetry transformations used to generate equivalent atoms:

#1 -x+1,y,-z+1/2

Anisotropic displacement parameters (Å^{2}x 10^3) for a. The anisotropic displacement factor exponent takes the form: $-2\pi^2[h^2a^{*2}U^{11} + ... + 2 h k a^* b^* U^{12}]$

	U^{11}	U^{22}	U^{33}	U^{23}	U^{13}	U^{12}
S(1)	16(1)	15(1)	21(1)	0(1)	-3(1)	-1(1)
F(1)	19(1)	34(2)	257(6)	23(2)	-33(2)	-4(1)

F(2)	33(1)	20(1)	42(1)	9(1)	-14(1)	-6(1)
F(3)	201(4)	65(2)	32(2)	2(1)	-28(2)	-83(3)
O(1)	89(3)	32(2)	27(2)	-11(1)	11(2)	-22(2)
O(2)	19(1)	33(2)	84(2)	18(2)	5(1)	6(1)
O(3)	25(1)	18(1)	51(2)	11(1)	-14(1)	-1(1)
N(1)	10(1)	14(1)	17(1)	-1(1)	-2(1)	2(1)
N(2)	16(1)	16(1)	17(1)	-2(1)	-1(1)	2(1)
C(1)	28(2)	14(2)	27(2)	-6(1)	-1(2)	5(1)
C(2)	32(2)	19(2)	30(2)	-10(2)	-2(2)	-2(2)
C(3)	21(2)	26(2)	15(2)	-4(1)	-1(1)	-6(1)
C(4)	16(2)	19(2)	12(1)	0(1)	2(1)	0(1)
C(5)	13(1)	15(2)	10(1)	0(1)	4(1)	0(1)
C(6)	15(2)	19(2)	31(2)	-4(1)	-6(1)	5(1)
C(7)	10(1)	12(2)	16(2)	0(1)	-1(1)	2(1)
C(8)	15(2)	16(2)	20(2)	2(1)	1(1)	-3(1)
C(9)	23(2)	13(2)	26(2)	3(1)	-2(2)	-2(1)
C(10)	29(2)	20(2)	36(2)	7(2)	-17(2)	-6(2)

Hydrogen coordinates (x 10^4) and isotropic displacement parameters (Å^{2}x 10^3)

for a.

	x	y	z	U(eq)
H(1)	3519	8025	3488	28
H(2)	4788	8419	4330	32

H(3)	5787	7029	4623	25
H(4)	5484	5294	4094	19
H(6A)	3111	6002	1955	32
H(6B)	2660	6962	2580	32
H(6C)	2611	5759	3019	32
H(7)	5056	3725	3628	15
H(8A)	3762	2710	3581	20
H(8B)	3774	2723	2333	20
H(9A)	5016	1646	3620	25
H(9B)	4309	1056	2915	25
H(5)	3670(30)	4570(30)	2660(30)	24(10)

Torsion angles [A°] for a.

C(5)-N(2)-C(1)-C(2)	1.8(5)
C(6)-N(2)-C(1)-C(2)	-176.7(3)
N(2)-C(1)-C(2)-C(3)	-1.6(5)
C(1)-C(2)-C(3)-C(4)	-0.5(5)
C(2)-C(3)-C(4)-C(5)	2.3(5)
C(7)-N(1)-C(5)-N(2)	171.9(3)
C(7)-N(1)-C(5)-C(4)	-6.3(5)
C(1)-N(2)-C(5)-N(1)	-178.2(3)
C(6)-N(2)-C(5)-N(1)	0.3(4)
C(1)-N(2)-C(5)-C(4)	0.0(4)
C(6)-N(2)-C(5)-C(4)	178.6(3)
C(3)-C(4)-C(5)-N(1)	176.1(3)

C(3)-C(4)-C(5)-N(2)	-2.1(4)
C(5)-N(1)-C(7)-C(8)	153.4(3)
C(5)-N(1)-C(7)-C(7)#1	-85.7(4)
N(1)-C(7)-C(8)-C(9)	-179.1(3)
C(7)#1-C(7)-C(8)-C(9)	58.3(4)
C(7)-C(8)-C(9)-C(9)#1	-56.5(4)
O(3)-S(1)-C(10)-F(3)	59.4(4)
O(1)-S(1)-C(10)-F(3)	-176.1(3)
O(2)-S(1)-C(10)-F(3)	-59.5(4)
O(3)-S(1)-C(10)-F(1)	-59.6(4)
O(1)-S(1)-C(10)-F(1)	64.9(4)
O(2)-S(1)-C(10)-F(1)	-178.4(3)
O(3)-S(1)-C(10)-F(2)	-177.8(3)
O(1)-S(1)-C(10)-F(2)	-53.2(3)
O(2)-S(1)-C(10)-F(2)	63.4(3)

Symmetry transformations used to generate equivalent atoms:

#1 -x+1,y,-z+1/2

Hydrogen bonds for a [Å and °].

D-H...A	d(D-H)	d(H...A)	d(D...A)	<(DHA)
C(1)-H(1)...F(3)#2	0.95	2.51	3.233(4)	132.7
C(2)-H(2)...F(1)#3	0.95	2.36	3.165(5)	142.4
C(4)-H(4)...O(2)#4	0.95	2.31	3.221(4)	160.4

C(6)-H(6B)...O(1)#5	0.98	2.58	3.442(5)	147.4
C(6)-H(6C)...F(3)	0.98	2.50	3.450(5)	163.9
N(1)-H(5)...O(1)#6	0.84(4)	2.45(4)	3.271(5)	167(4)

Symmetry transformations used to generate equivalent atoms:

#1 -x+1,y,-z+1/2 #2 -x+1/2,y+1/2,z #3 x+1/2,-y+3/2,-z+1

#4 -x+1,-y+1,-z+1 #5 -x+1/2,-y+3/2,z-1/2 #6 x,-y+1,z-1/2
